# Evaluating protein cross-linking as a therapeutic strategy to stabilize SOD1 variants in a mouse model of familial ALS

**DOI:** 10.1371/journal.pbio.3002462

**Published:** 2024-01-30

**Authors:** Md Amin Hossain, Richa Sarin, Daniel P. Donnelly, Brandon C. Miller, Alexandra Weiss, Luke McAlary, Svetlana V. Antonyuk, Joseph P. Salisbury, Jakal Amin, Jeremy B. Conway, Samantha S. Watson, Jenifer N. Winters, Yu Xu, Novera Alam, Rutali R. Brahme, Haneyeh Shahbazian, Durgalakshmi Sivasankar, Swathi Padmakumar, Aziza Sattarova, Aparna C. Ponmudiyan, Tanvi Gawde, David E. Verrill, Wensheng Yang, Sunanda Kannapadi, Leigh D. Plant, Jared R. Auclair, Lee Makowski, Gregory A. Petsko, Dagmar Ringe, Nathalie Y. R. Agar, David J. Greenblatt, Mary Jo Ondrechen, Yunqiu Chen, Justin J. Yerbury, Roman Manetsch, S. Samar Hasnain, Robert H. Brown, Jeffrey N. Agar

**Affiliations:** 1 Department of Chemistry and Chemical Biology, Northeastern University, Boston, Massachusetts, United States of America; 2 Barnett Institute of Chemical and Biological Analysis, Boston, Massachusetts, United States of America; 3 Department of Neurosurgery and Department of Radiology, Brigham and Women’s Hospital, Harvard Medical School, Boston, Massachusetts, United States of America; 4 Biogen Inc, Cambridge, Massachusetts, United States of America; 5 Department of Neurology, University of Massachusetts Medical School, Worcester, Massachusetts, United States of America; 6 Molecular Horizons and School of Chemistry and Molecular Bioscience, University of Wollongong, Wollongong, Australia; 7 Molecular Biophysics Group, Department of Biochemistry & Systems Biology, Faculty of Health and Life Sciences, University of Liverpool, Liverpool, United Kingdom; 8 Department of Pharmaceutical Sciences, Northeastern University, Boston, Massachusetts, United States of America; 9 School of Medicine, University of North Carolina, Chapel Hill, North Carolina, United States of America; 10 Department of Bioengineering, Northeastern University, Boston, Massachusetts, United States of America; 11 Ann Romney Center for Neurologic Diseases at Brigham and Women’s Hospital, Harvard Medical School, Boston, Massachusetts, United States of America; 12 Departments of Chemistry and Biochemistry, and Rosenstiel Center for Basic Medical Research, Brandeis University, Waltham, Massachusetts, United States of America; 13 Department of Cancer Biology, Dana-Farber Cancer Institute, Harvard Medical School, Boston, Massachusetts, United States of America; 14 School of Medicine, Tufts University, Boston, Massachusetts, United States of America; Stony Brook University Medical Center: Stony Brook University Hospital, UNITED STATES

## Abstract

Mutations in the gene encoding Cu-Zn superoxide dismutase 1 (SOD1) cause a subset of familial amyotrophic lateral sclerosis (fALS) cases. A shared effect of these mutations is that SOD1, which is normally a stable dimer, dissociates into toxic monomers that seed toxic aggregates. Considerable research effort has been devoted to developing compounds that stabilize the dimer of fALS SOD1 variants, but unfortunately, this has not yet resulted in a treatment. We hypothesized that cyclic thiosulfinate cross-linkers, which selectively target a rare, 2 cysteine-containing motif, can stabilize fALS-causing SOD1 variants in vivo. We created a library of chemically diverse cyclic thiosulfinates and determined structure-cross-linking-activity relationships. A pre-lead compound, “*S*-XL6,” was selected based upon its cross-linking rate and drug-like properties. Co-crystallographic structure clearly establishes the binding of *S*-XL6 at Cys 111 bridging the monomers and stabilizing the SOD1 dimer. Biophysical studies reveal that the degree of stabilization afforded by *S*-XL6 (up to 24°C) is unprecedented for fALS, and to our knowledge, for any protein target of any kinetic stabilizer. Gene silencing and protein degrading therapeutic approaches require careful dose titration to balance the benefit of diminished fALS SOD1 expression with the toxic loss-of-enzymatic function. We show that *S*-XL6 does not share this liability because it rescues the activity of fALS SOD1 variants. No pharmacological agent has been proven to bind to SOD1 in vivo. Here, using a fALS mouse model, we demonstrate oral bioavailability; rapid engagement of SOD1^G93A^ by *S*-XL6 that increases SOD1^G93A^’s in vivo half-life; and that *S*-XL6 crosses the blood–brain barrier. *S*-XL6 demonstrated a degree of selectivity by avoiding off-target binding to plasma proteins. Taken together, our results indicate that cyclic thiosulfinate-mediated SOD1 stabilization should receive further attention as a potential therapeutic approach for fALS.

## Abstract 2

Mutations in the Cu-Zn superoxide dismutase 1 gene SOD1 can cause familial amyotrophic lateral sclerosis (fALS) in a process that involves dissociation of the SOD1 dimer. This study shows that a novel cyclic thiosulfinate cross-linker has favorable drug-like properties and can stabilize the SOD1 dimer in vivo, with therapeutic implications for fALS.

## Introduction

Amyotrophic lateral sclerosis (ALS) is a fatal neurodegenerative disease that often leads to death within 2 to 5 years of diagnosis [1,2], and 5% to 10% of ALS cases are familial (fALS) while the rest are sporadic [3,4]. Over 180 mutations in the gene encoding superoxide dismutase-1 (SOD1) contribute to approximately 20% of fALS cases [5,6]. These mutations are generally dominantly inherited, highly penetrant, and associated with varying degrees of disease severity and loss of enzymatic activity [7,8]. For example, patients harboring the SOD1 variant SOD1^A4V^ do not often survive more than 1 year after diagnosis, whereas those with SOD1^H46R^ survive an average of 18 years [9]. Many studies have also implicated *wild-type* SOD1 in idiopathic, sporadic ALS (sALS). For example, posttranslational modifications (PTMs) such as oxidation of Cys111 [10] are toxic and autoantibodies [11] to SOD1 are more prevalent in sALS cases. However, the role of *wild-type* SOD1 in ALS remains a subject of debate [12]. The cyclic thiosulfinates used in this study block oxidation of Cys111 and could be a useful chemical tool for addressing the role of wild-type SOD1 in sALS.

*Wild-type* SOD1 is a homodimer. A common property of ALS-associated SOD1 mutations is that they increase the propensity of the dissociation of the SOD1 dimer, which promotes misfolding and aggregation of the resulting monomers into cytotoxic species [13]. Small molecules are therefore being developed to stabilize the quaternary structure of fALS SOD1 variants [14,15]. An analogous strategy was employed successfully to create tafamidis, a drug for transthyretin amyloidosis and transthyretin cardiomyopathy, which stabilizes the native transthyretin tetramer [16]. Unfortunately, molecules intended to stabilize SOD1 via non-covalent interactions at the dimer interface [17] instead bound to the β-barrel region of the SOD1 aggregates [14] and often exhibited plasma protein binding [15].

Here, we pursue an alternative approach to stabilize the SOD1 dimer. We postulate that 2 free cysteines situated on adjacent monomers, Cys111_a_ and Cys111_b_ (separate by approximately 9 Å), could be cross-linked to prevent dimer dissociation. Cysteine residues can be targeted with high selectivity due to the unique reactivity (nucleophilicity and polarizability) of the thiolate functional group. This is evidenced by numerous drugs that form covalent bonds to cysteine residues, including the irreversible aldehyde dehydrogenase (ALDH1A1) inhibitor disulfiram (Antabuse) [18]; the blockbuster proton pump inhibitors, e.g., omeprazole (Prilosec) and its single enantiomer esomeprazole (Nexium) [19]; inhibition of Severe Acute Respiratory Syndrome Coronavirus 2 (SARS-CoV-2) main protease by ebselen [20] and Paxlovid, and second-generation kinase inhibitors, e.g., afatinib (Gilotrif) [21]. Formation of covalent bonds to cysteines has also been shown to provide access to “undruggable” targets, as evidenced by Amgen’s drug for KRAS G12C mutations, sotorasib. In proof-of-concept experiments, we demonstrated that cross-linking the Cys111_a,b_ pair on the disease variants SOD1^G93A^ and SOD1^G85R^ could stabilize the SOD1 dimer and rescue superoxide dismutase activity [22]. The bifunctional maleimide cross-linkers used in these studies, however, are toxic [23] because they target any exposed free cysteine, many of which serve essential catalytic and redox roles for other proteins. Rather than cross-linking, ebselen and cisplatin target SOD1 Cys111 [24,25] and stabilize the SOD1 dimer by non-covalent interactions. Ebselen has been shown to delay onset and improve survival in the G93A mouse model [26]. These promising preclinical leads may share maleimide’s propensity for off-target binding, as evidenced by ebselen’s off-target binding to Cys6 [27] and the hepatotoxicity of cisplatin. A therapeutically viable cross-linking mechanism for SOD1-dependent fALS should bind the Cys111 pair while avoiding other cysteines.

We recently introduced cyclic thiosulfinates, which selectively cross-link pairs of free cysteines, while avoiding “dead-end” modifications of lone cysteines [28]. The cyclic thiosulfinate 1,2-dithiane-1-oxide (*S*ulfur cross-linking *6*-membered ring, hereafter *S*-XL6) and β-lipoic acid (the *S*-oxo derivative of α-lipoic acid) [28], target the Cys111_a_ and Cys111_b_ residues on adjacent SOD1 monomers, forming a disulfide bond with each cysteine thiolate. Cyclic thiosulfinates are one oxygen (*S*-oxo) derivatives of cyclic disulfides. Well-known and well-tolerated cyclic disulfides include the natural product asparagusic acid and the multi-enzyme cofactor and dietary supplement α-lipoic acid. In this study, we test the following hypotheses: (1) cross-linking improves the biochemical properties of fALS SOD1 variants; and (2) *S*-XL6 engages SOD1 in vivo.

## Results

**Fig 1:**
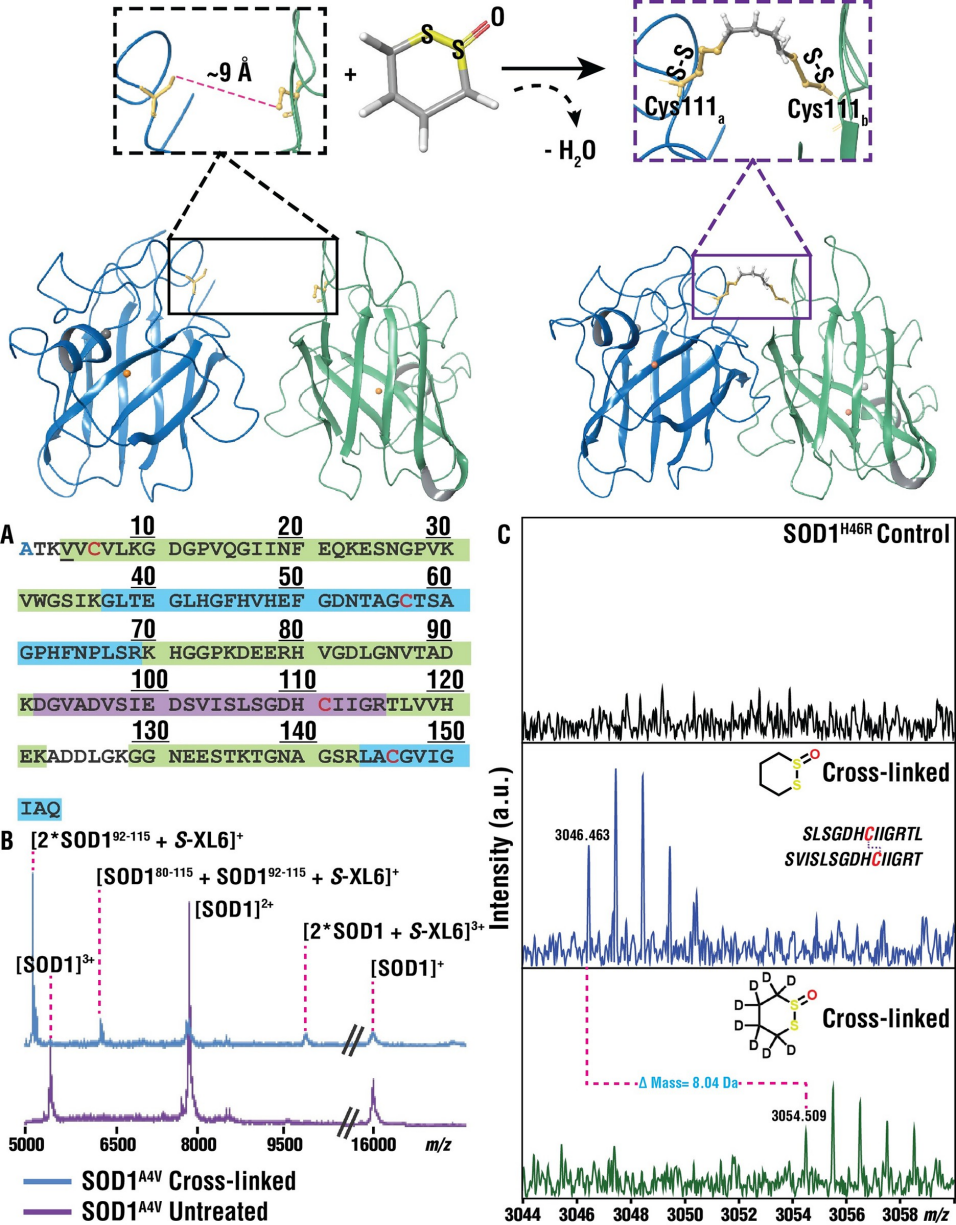
Cyclic thiosulfinate *S*-XL6 cross-links SOD1 variants via Cys111 residues on adjacent monomers. ** (Top)** Crystal structure of *wild-type* SOD1 (PDB ID: 1SPD [5], cartoon representation generated with Maestro 11.8) highlighting opposing Cys111 residues on both monomer A (blue) and monomer B (green) with a representation of the *S*-XL6 cross-linked SOD1 dimer. Cu and Zn molecules are represented by orange and gray spheres, respectively. (**A**) Trypsin digest and MALDI-ToF-MS analysis of SOD1^A4V^ gave a combined sequence coverage of 94%, including peaks corresponding to the Cys57-Cys146 disulfide linked peptides (blue) and the Cys111- containing peptide (purple) linked by *S*-XL6. N-terminal acetylation is shown in blue text, cysteines are shown in red text, and the SOD1^A4V^ mutation is underlined. (**B**) Examining the higher mass range revealed multiply charged forms of SOD1^A4V^ including the *S*-XL6 linked dimer, as well as 2 different peaks corresponding to Cys111-linked peptides. (**C**) MALDI-FTICR-MS analysis of pepsin digested SOD1^H46R^. The top panel shows SOD1^H46R^ control sample and middle panel exhibits peaks corresponding to Cys111 linked peptides via *S*-XL6. The cross-linked peptides are shown in black text with cysteines highlighted in red. The bottom panel shows confirmation of cross-linked peptides using deuterated *S*-XL6 (mass shift of 8 Da). MALDI, matrix-assisted laser desorption ionization; MS, mass spectrometry; SOD1, superoxide dismutase 1.

A computational search was performed in PubChem to identify all compounds containing a cyclic disulfide motif. These compounds, which consisted of 186 cyclic disulfide derivatives, were acquired from the National Cancer Institute (NCI) and screened for the ability to cross-link SOD1. Of these, 69 compounds cross-linked SOD1 with a wide array of reaction rates. Only 2 compounds could cross-link 100% of SOD1 during the assay, including one 5-membered cyclic disulfide (4-Amino-1,2-Dithiolane-4-Carboxylic Acid) and one 6-membered cyclic thiosulfinate (1,2-dithiane 1-oxide). Additional cyclic thiosulfinates were synthesized to create series and fill gaps in chemical space, including 5- (1,2-dithiolane 1-oxide), 6- (1,2-dithiane 1-oxide), 7- (1,2-dithiepane 1-oxide), and 8-membered rings (1,2-dithiocane 1-oxide) and their properties were determined (e.g., EC_50_ for cross-linking SOD1, cytotoxicity, solubility, clearance, cross-linking rate, LD_50_ in mice). The results of these studies—in particular, the difference between EC_50_ and LD_50_—suggested that only the 5- and 6-membered rings had potential as fALS therapeutics. The 6-membered cyclic thiosulfinate, 1,2-dithiane 1-oxide (*S*-XL6, **Fig 1**), by virtue of its scalable synthesis and lower cytotoxicity and clearance, was chosen for initial characterization.

To test the efficacy of cross-linker-mediated SOD1 stabilization, we chose fALS variants ranging from “wild-type-like” (metalated and enzymatically active SOD1^A4V^ and SOD1^G93A^), to “metal deficient” (impaired metal binding and inactive SOD1^H46R^ and SOD1^G85R^). These variants are aggregation-prone, span a wide range of clinical prognoses (1 year to 18 years survival) and have a range of properties that is believed to represent most fALS variants. Using these, we tested the hypothesis that cross-linking improves the biochemical/biophysical properties of fALS SOD1 variants, e.g., that *S*-XL6 increases thermal stability and restores structure and enzymatic activity. We then tested the hypothesis that cross-linking can stabilize a fALS SOD1 variant in vivo and improve survival.

### *S*-XL6 cross-links and stabilizes fALS SOD1 variants with diverse physicochemical properties

**Fig 2:**
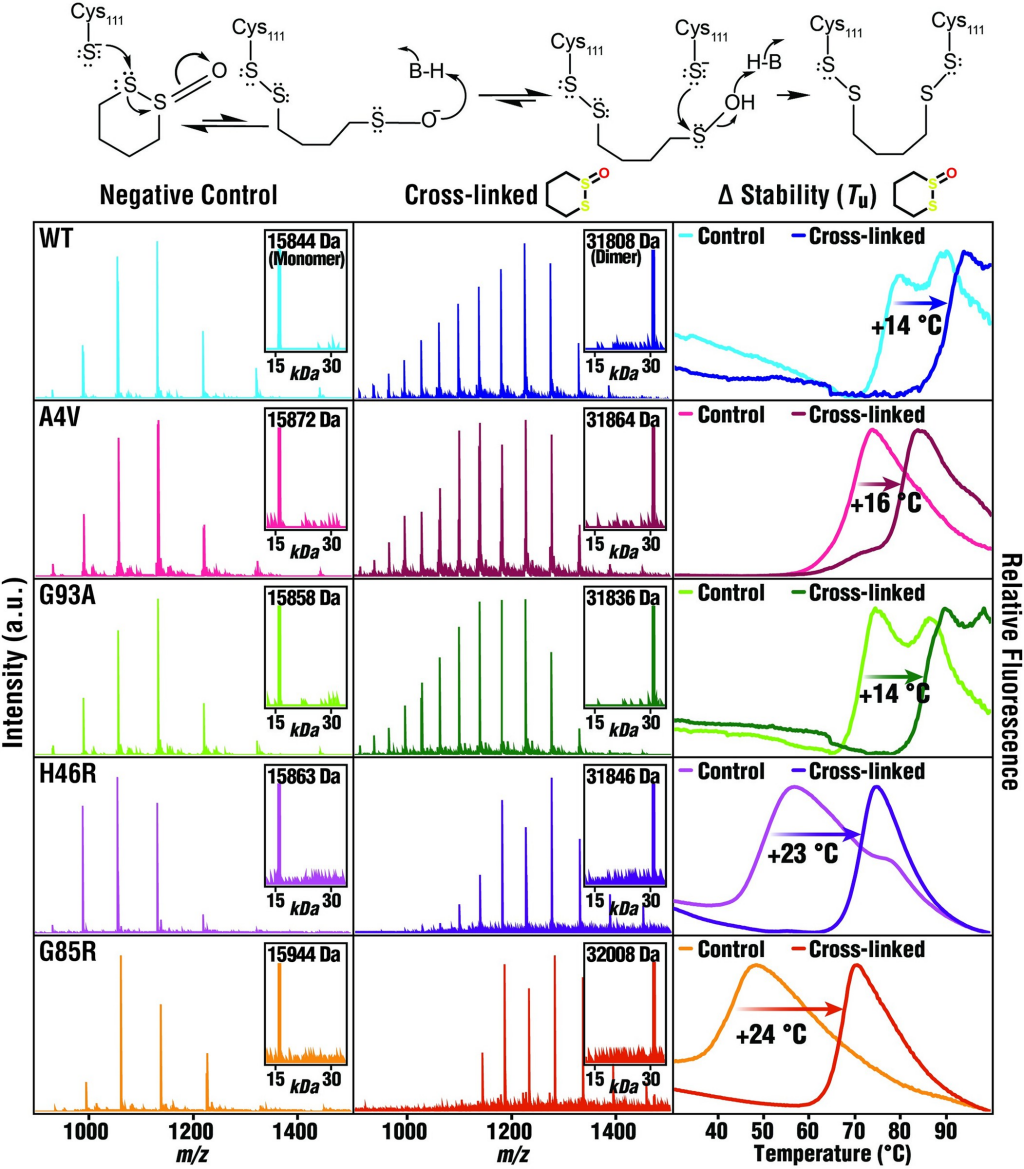
*S*-XL6-mediated cross-linking and stabilization of *wild-type* and fALS SOD1 variants. Top: chemical mechanism of action of S-XL6. The cross-linking reaction proceeds through an initial thiolate-disulfide interchange between a cysteine thiolate and the cyclic thiosulfinate, generating a sulfenic acid intermediate upon opening of the ring structure. This sulfenic acid intermediate forms a cross-link by rapid condensation with a second cysteine thiolate. Mass spectra of untreated (**left column,** average mass [± 1 Da]) and *S*-XL6 cross-linked (**middle column,** average mass [± 2 Da]) proteins are consistent with cross-linking. DSF results (**right column**) indicate that cross-linking increased the thermal stability of SOD1 and its variants. We quantify Δ*T*_u_ as the difference in unfolding temperature measured between the major inflections of the untreated and cross-linked samples. The unfolding temperature of the untreated and *S*-XL6 cross-linked SOD1 proteins are as follows: *wild type* (WT) from 75.9°C to 90.2°C (Δ*T*_u_ ~14°C); SOD1^A4V^ from 62.9°C to 79.3°C (Δ*T*_u_ ~16°C); SOD1^G93A^ from 70.6°C to 85.0°C (Δ*T*_u_ ~14°C); SOD1^H46R^ from 46.3°C to 69.3°C (Δ*T*_u_ ~23°C); SOD1^G85R^ from 40.8°C to 65.1°C (Δ*T*_u_ ~24°C). Note: two inflections can be observed when there is a mixture of partially (lower Δ*T*_u_) and fully metalated (higher Δ*T*_u_) SOD1 proteoforms, which applies to the following proteins: untreated *wild-type* SOD1 unfolds at 75.9°C and 87.6°C; untreated SOD1^G93A^ unfolds at 70.6°C and 84.4°C. All samples were analyzed in technical triplicate (standard errors <0.3°C). fALS, familial amyotrophic lateral sclerosis; SOD1, superoxide dismutase 1.

We employed a mass spectrometry (MS) assay for intact protein cross-linking [22,29]. In this assay, any non-covalent (i.e., native) dimer is dissociated by a combination of acidic, organic media and MS source conditions. Using this assay, we observed *S*-XL6-dependent formation of cross-linked dimers for *wild-type* SOD1, SOD1^A4V^, SOD1^G93A^, SOD1^H46R^, and SOD1^G85R^ (**Fig 2, left**). This reaction was efficient (full conversion of monomer to dimer) and stoichiometric (occurring at ratios as low as 1:1 cross-linker to dimer). Dead-end modifications of SOD1 were not observed in this assay. We confirmed the mechanism of action (MoA, e.g., loss of oxygen from the cross-linker concomitant with dimer formation, **Fig 1**) and determined the site of cross-linking using endoproteinase digestion and MALDI (matrix-assisted laser desorption ionization) peptide mass fingerprinting analysis of cross-linked samples (**Fig 1**).

SOD1 mutations destabilize the native dimer to varying degrees. Loss of stability of fALS variants correlates with disease severity, specifically with rapid progression [30]. To understand how cross-linking effects SOD1 stability, we quantified changes in unfolding temperature (Δ*T*_u_, hereafter thermal stability). Purified *wild-type*, SOD1^A4V^, SOD1^G93A^, SOD1^H46R^, and SOD1^G85R^ proteins were treated with *S*-XL6. Thermally induced unfolding of SOD1 variants was measured using a previously published method, differential scanning fluorimetry (DSF) (**Fig 2, right**) [31]. The increases in thermal stability attained with *S*-XL6 (14 to 24°C) compare favorably with the best preclinical candidate for stabilizing SOD1 (e.g., 11°C for ebselen analogues, [26]) and with the only approved drug with a kinetic stabilizing mechanism, tafamidis (6°C) [32].

### Structural analysis of *S*-XL6 cross-linked fALS SOD1 variants

**Fig 3:**
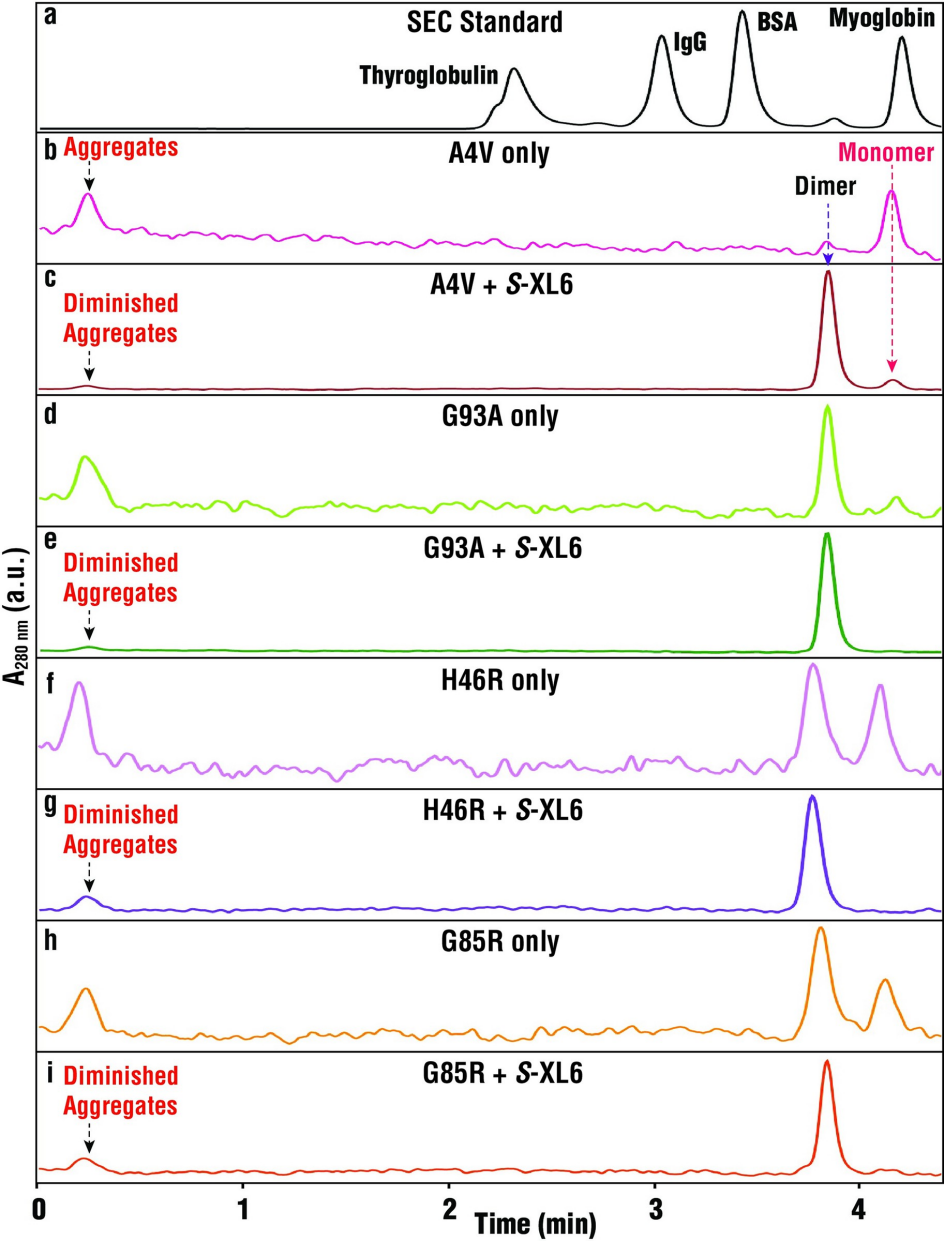
SEC demonstrates that the monomeric population of fALS variants is decreased by *S*-XL6 treatment and that this is associated with reduced aggregation. Top: SEC results for the molecular weight calibration standards, Thyroglobulin (660 kDa), IgG (150 kDa), BSA (66 kDa), and myoglobin (17 kDa). Bottom: SEC results for the same variants as Figs 2 and 4, with and without *S*-XL6 treatment. The peak labeled “aggregates” elute near the void volume of the SEC column and are therefore too large (>1,000 kDa) for MW determination. fALS, familial amyotrophic lateral sclerosis; SEC, size exclusion chromatography.

To determine the impact of cross-linking on the higher order structure of fALS SOD1 variants, we utilized size exclusion chromatography (SEC), hydrogen deuterium exchange mass spectrometry (H/D-X MS), X-ray crystallography, and small-angle X-ray scattering (SAXS). Whereas the MS assay above was performed under denaturing conditions, SEC was performed under native conditions. SEC studies confirmed previous research showing that a substantial proportion of fALS variants are monomeric near physiological concentration and would benefit from kinetic stabilization (**Figs 3 and S9**). The SEC results also confirmed the conversion of fALS SOD1 monomers into dimer following *S*-XL6 treatment and provide a complementary measurement of cross-linking efficiency. Whereas MS-based detection is heavily biased towards low molecular mass species (i.e., generally cannot detect large aggregates) SEC can detect large oligomers [14,33–37]. The SEC results show that large (>1,000 kDa) aggregates are present in all preparations of untreated fALS variants. Notably, these aggregates were diminished in all the fALS variants we tested following treatment with *S*-XL6. The ability of *S*-XL6 to diminish aggregates is expected based upon *S*-XL6-mediated stabilization of SOD1 variants, given that stabilization increases the time required for the nucleation phase of aggregation. We are currently unable to measure the rates of aggregation because existing assays require reductants that would also remove *S*-XL6 [38,39].

**Fig 4:**
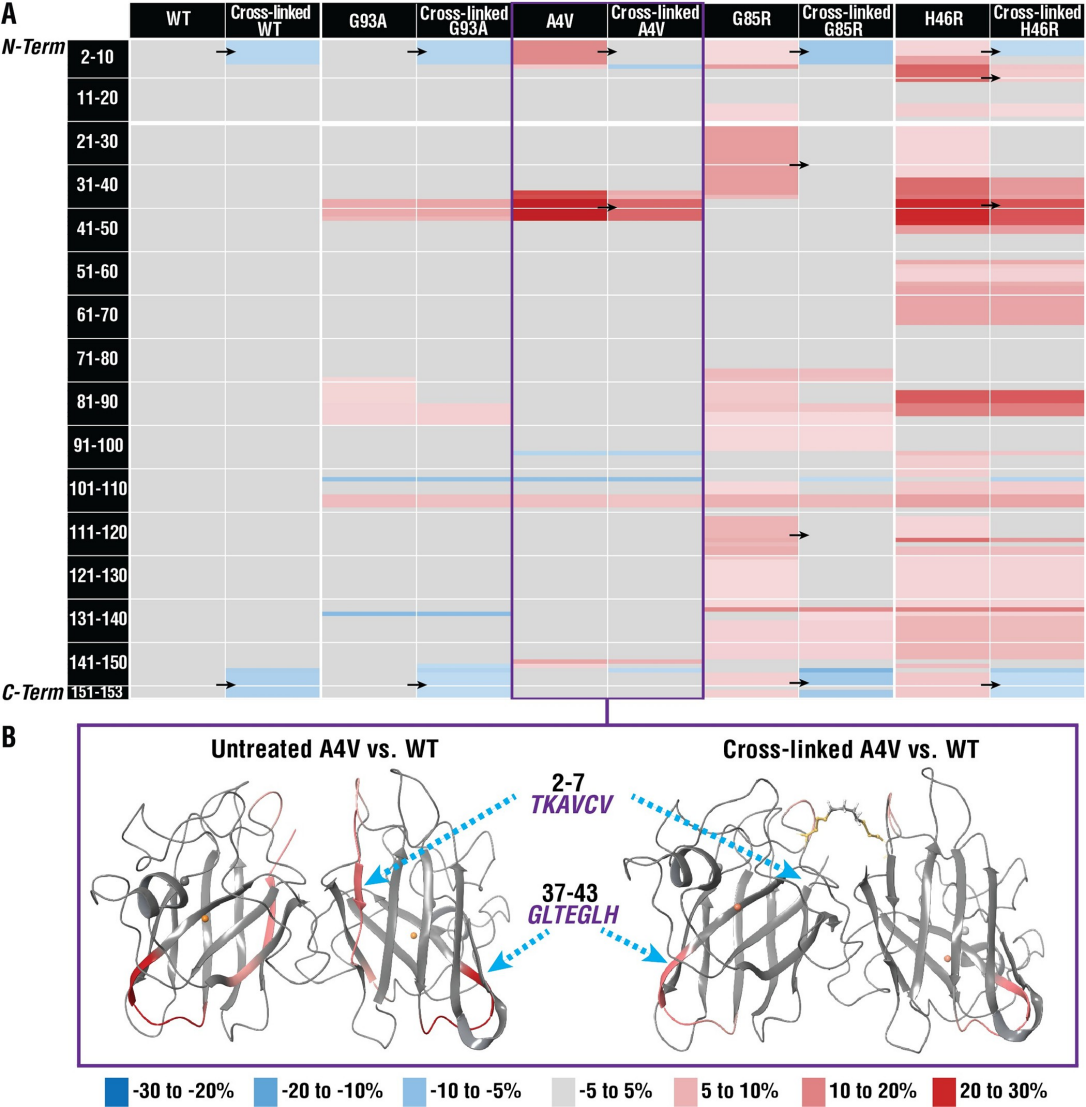
Cross-linking enhances the structure of fALS SOD1 variants. Differences in deuterium uptake (ΔU, legend shown below part b) of untreated and cross-linked variants for the 4-h time point compared to the *wild-type* SOD1 (WT) protein are reported here. **(A)** Black arrows indicate areas where prominent shifts in ΔU were observed (e.g., residues 2–7 for SOD1^A4V^ untreated 13.8% to 1.2% cross-linked, for full results see **S1 Fig**). **(B)** ΔU for untreated and cross-linked SOD1^A4V^ mapped onto the cartoon representation of *wild-type* SOD1 structure (PDB ID: 1SPD) [5], generated with Maestro 11.8. ΔU for all time points (15 s, 50 s, 500 s, 1 h, 4 h) are reported in **S1 Fig.** fALS, familial amyotrophic lateral sclerosis; SOD1, superoxide dismutase 1.

H/D-X MS assesses structure and dynamics by measuring differences in deuterium uptake [40]. Following cross-linking, we observed that the structures of fALS SOD1 variants bear a closer resemblance to, but do not fully recapitulate, the structure of *wild-type* SOD1 structure (**Fig 4**). For example, decreased uptake at the N- and C-termini of SOD1 (the dimer interface) was observed in all cross-linked variants. In all but one variant, SOD1^G93A^, the uptake around residues 37–43 more closely resembled *wild type*. Notably, structural changes in this region are responsible for the “gain of interaction” with the disordered electrostatic loop, which has been proposed to lead to the aggregation of fALS variants [41]. Previous studies have classified the enzymatically active SOD1^A4V^ and SOD1^G93A^ proteins [41,42] as *wild-type*-like variants and the inactive, less-folded SOD1^G85R^ and SOD1^H46R^ proteins [43,44] as metal-deficient variants. Consistent with this, larger differences in uptake via H/D-X MS were observed for SOD1^G85R^ and SOD1^H46R^. Subtle (<5%) differences in deuteration levels were also observed in other regions of the SOD1 variants. In summary, cross-linking makes the structure and dynamics of fALS SOD1 variants more like that of *wild-type* SOD1 and in a manner that is consistent with reducing their aggregation propensity.

### Enzymatic activity of cross-linked SOD1 variants is rescued to that of *wild-type* SOD1

**Fig 5:**
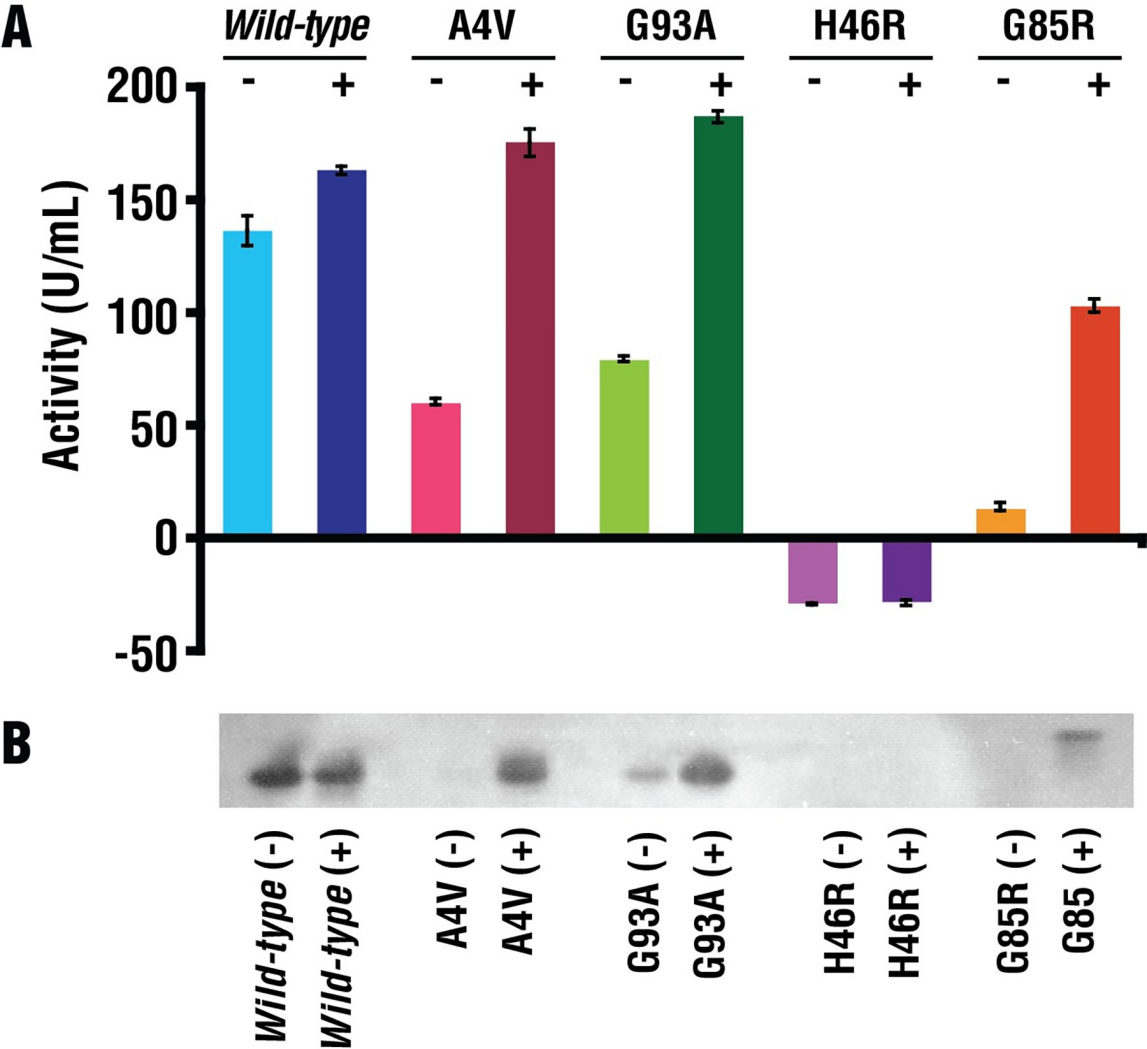
Enzymatic activity of SOD1 variants after *S*-XL6 treatment. Plate-based biochemical (**A**) and gel-based (**B**) assays were used to assess the impact of cross-linking on the enzymatic activity of SOD1 variants. Upon treatment with cross-linker the activity of SOD1^A4V^, SOD1^G93A^, and SOD1^G85R^ increased to that of *wild-type* SOD1. Treatment with crosslinker did not affect the enzymatically inactive SOD1^H46R^. “+” and “–” denote samples treated with *S*-XL6 and untreated samples, respectively. The data underlying this figure can be found in S1 Data. SOD1, superoxide dismutase 1.

Utilizing both plate-based (biochemical) and gel-based assays, we characterized the impact of cross-linking on the enzymatic activity of SOD1 variants. Cross-linking increased the activity of fALS SOD1^A4V^, SOD1^G93A^, and SOD1^G85R^ variants to levels comparable to that of *wild-type* SOD1 (**Fig 5A and *5*B and S1 Table**). No change in activity was observed for H46R, which was as expected because His46 is required for Cu binding and enzymatic activity. This result, combined with metal content in S4 Table indicating that variant have less Cu than WT-SOD1, and the requirement of Cu for enzymatic activity, imply that S-XL6 enables additional Cu incorporation into SOD1.

### A cross-linker with promising toxicological properties promotes SOD1 dimer formation in cellulo

**Fig 6:**
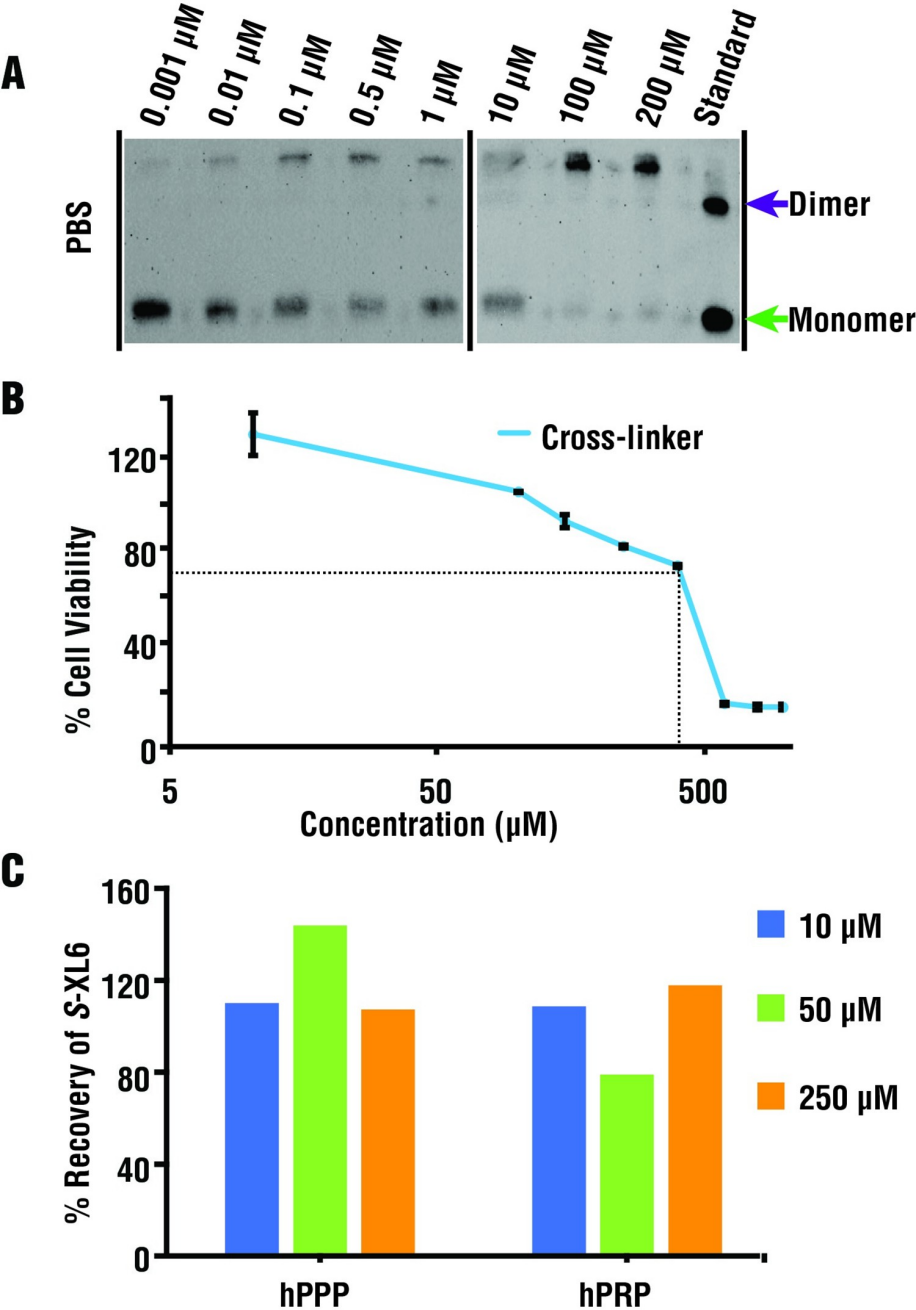
Cross-linking at sub-toxic concentrations within cells. **(A)** Western blot analysis using a SOD1-selective antibody indicates that cross-linking proceeds in HEP G2 cells with an EC_50_ of approximately 5 μm (in PBS buffer). **(B)** Low cytotoxicity of *S*-XL6 (LC_50_ approximately 446 μm) was observed using the MTT (3-(4,5-Dimethylthiazol 2-yl)-2,5-diphenyltetrazolium bromide) cytotoxicity assay (triplicate sample analysis). Results are shown as percentage of viable cells compared to a vehicle control. Note: cell viability <70% (dotted line) is generally considered as the threshold for cytotoxicity, and viabilities >100% indicate a trophic effect. Controls included EMEM with 0.1% DMSO (dimethyl sulfoxide, negative control) and 500 μm of chlorpromazine (positive control). **(C)** Plasma protein binding was assayed in human platelet rich (hPRP) and platelet poor plasma (hPPP) using an LC-MS/MS assay at 3 concentrations (10, 50, and 250 μm) and no binding of *S*-XL6 was observed. Likewise, *S*-XL6 was not bound to purified alpha 1-acid glycoprotein and human serum albumin. The data underlying this figure can be found in S1 Data. LC-MS/MS, liquid chromatography-tandem mass spectrometry; MS, mass spectrometry; SOD1, superoxide dismutase 1.

We characterized the effectiveness of our crosslinker using Hep G2 cells. SOD1 cross-linking was monitored using SDS-PAGE and western blotting. *S*-XL6 cross-linked *wild-type* SOD1 in cells in PBS buffer with the half maximal effective concentrations (EC_50_) at circa (*ca*.) 5 μm (**Fig 6A**). These results are also consistent with previous research indicating that cyclic thiosulfinates are actively transported across the cellular membrane [28] where they remain intact [45]. Plasma protein binding was directly assessed using human plasma and no binding of *S*-XL6 was observed (**Fig 6C**). The minimal plasma protein binding is promising and is enabled by cyclic thiosulfinates’ ability to avoid lone cysteines, including the highly abundant and reactive lone cysteine (Cys34) [46] of serum albumin.

**Fig 7:**
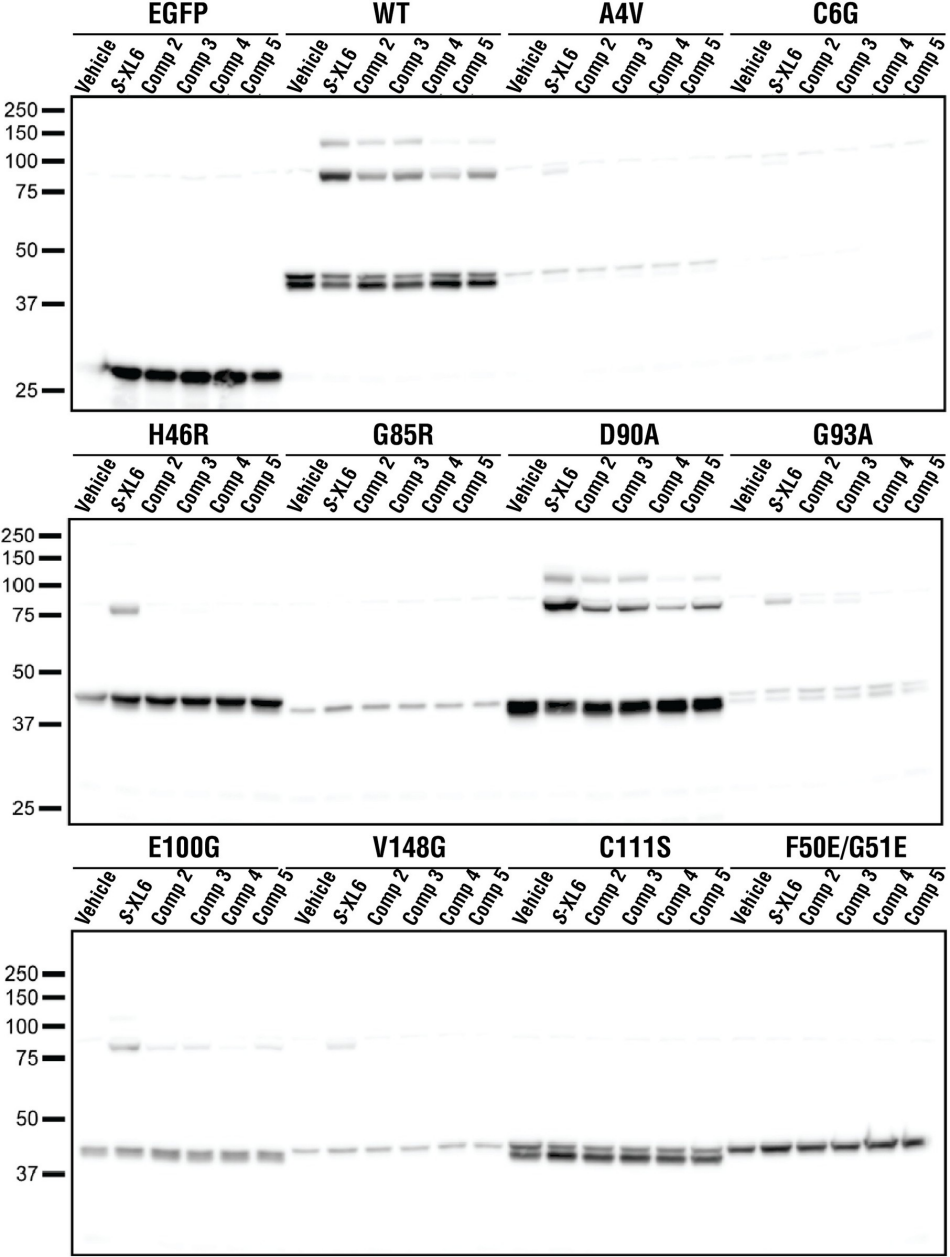
*S*-XL6 outperforms leading cyclic thiosulfinates, binds a variety of fALS variants, and requires the Cys111 residue in cellulo. C-terminally EGFP-labeled SOD1 (wild-type SOD1, 8 fALS variants, a Cys111Ser variant, and a constitutively monomeric F50E/G51E variant) were expressed in NSC-34 cells. *S*-XL6 was the most effective compound of the top-five 6-membered cyclic thiosulfinates identified through mass spectrometry screening of NCI compounds and our own medicinal chemistry efforts (labeled “Comp 2–5”). Compounds **2** and **3** are epimers of the 4,5-hydroxyl derivates of *S*-XL6; the identity of compounds 4 and 5 are unpatented and cannot be disclosed here, and 20 μm *S*-XL6 engaged all fALS variants other than G85R and wild-type SOD1, but not Cys111Ser or F50E/G51E. EGFP-wild-type and EGFP-D90A SOD1 formed an off-product (unintended) high-molecular weight species consistent with trimeric SOD1. S-XL6 treatment did not affect the cellular viability or aggregation of G93A SOD1. *S*-XL6 treatment did not affect the viability but did accelerate the aggregation of wild-type SOD1 (S3 **Fig**). fALS, familial amyotrophic lateral sclerosis; SOD1, superoxide dismutase 1.

The cytotoxicity of *S*-XL6 was measured in Hep G2 using a standard MTT assay (LC_50_ approximately 446 μm, **Fig 6B**). Given that the LC_50_ is 90× the EC_50_, *S*-XL6 can promote dimer formation in cellulo with minimal toxicity. *S*-XL6 did not affect the survival or aggregation of EGFP-labeled G93A in NSC-34 cells (S3 **Fig**). *S*-XL6 treatment of EGFP-labeled wild-type SOD1 increased cellular aggregation and a high-molecular weight species observed in western blots in NSC-34 cells (S3 **Fig**). We note that the same high-molecular weight species were not observed in HEPG2 (**Fig 6**) or HELA cells [28], which natively express wild-type SOD1. Further studies will determine if S-XL6-dependent inclusion formation of wild-type SOD1 is neuron-related or EGFP-labeling-related. Results obtained in NSC-34 cells, specifically the lack of cross-linking in the Cys111Ser variant, indicate that the Cys111 residue is targeted by S-XL6 and is required for cross-linking. Results obtained in NSC-34, specifically the lack of binding to a constitutively monomeric variant, also indicate that the dimeric subpopulation of variants is targeted. The binding of *S*-XL6 to a wider array of fALS variants (EGFP-labeled) was also tested in NSC-34 cells and was compared to that of our best third-generation compounds. Compared to our leading compounds, *S*-XL6 consistently resulted in the highest cross-linking yield and was therefore chosen for in vivo studies (**Fig 7**).

### *S*-XL6 binds to Cys111 and cross-links the monomers increasing dimer stability

**Fig 8:**
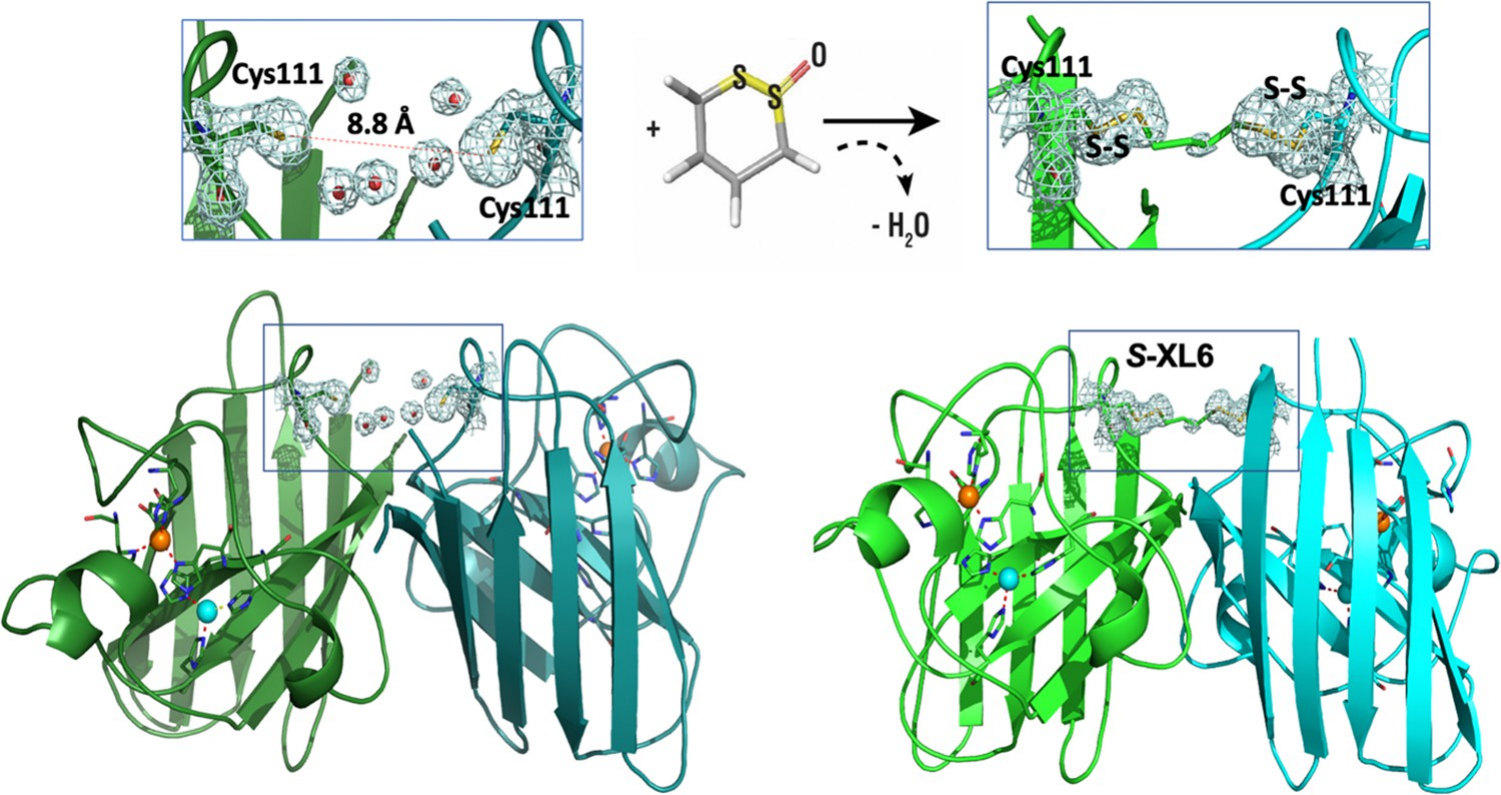
Cyclic thiosulfinate *S*-XL6 cross-links *wild-type* SOD1 via Cys111 residues on adjacent monomers. On the left crystal structure of as-isolated *wild-type* SOD1 at 1.77 Å resolution (cartoon representation generated with Pymol) highlighting opposing Cys111 residues on both monomer A (dark green) and monomer B (dark blue) with multiple water molecule present at dimer interface (PDB ID 8Q6M). Right hand panels show the crystal structure of *S*-XL6 cross-linked *wild-type* SOD1 at 1.67Å resolution (PDB ID 8CCX) clearly displaying additional density for S atoms from *S*-XL6 on both monomers A (light green) and B (cyan). 2FoFc electron density maps for the structures are shown as gray mesh, contoured at 1 σ level around Cys111, water molecules at the SOD1 dimer interface and cross-linker. Waters are indicated as small red spheres, Cu and Zn molecules are represented by cyan and orange spheres, respectively. These water molecules are largely excluded upon the incorporation of *S*-XL6. The data underlying this figure can be found at rcsb.org (PDB IDs 8Q6M and 8CCX). SOD1, superoxide dismutase 1.

Co-crystallographic structure of SOD1 soaked with S-XL6 was determined to 1.67 Å resolution using the X-ray facility at the Barkla X-ray laboratory of Biophysics, Liverpool. As a control, as-isolated SOD1 structure was also determined under a similar low-dose conditions to 1.77 Å resolution. Resolution of the as-isolated structure is a little less as a smaller crystal was used for this purpose. The high-resolution structure of the complex gives clear evidence for the binding of the compound at Cys111 while the as-isolated structure shows a number of water molecules (**Fig 8**). Specifically, the structure of SOD1 with S-XL6 shows that the linker is attached by S-S bridges to SD atoms of Cys111 and connects 2 monomers (**Fig 8**). The electron density for carbon atoms of the compound is much weaker than for sulphur atoms, probably due to the flexibility of carbon chain and/or partial opening of the ring structure due to a water-mediated hydrolysis [20].

**Fig 9:**
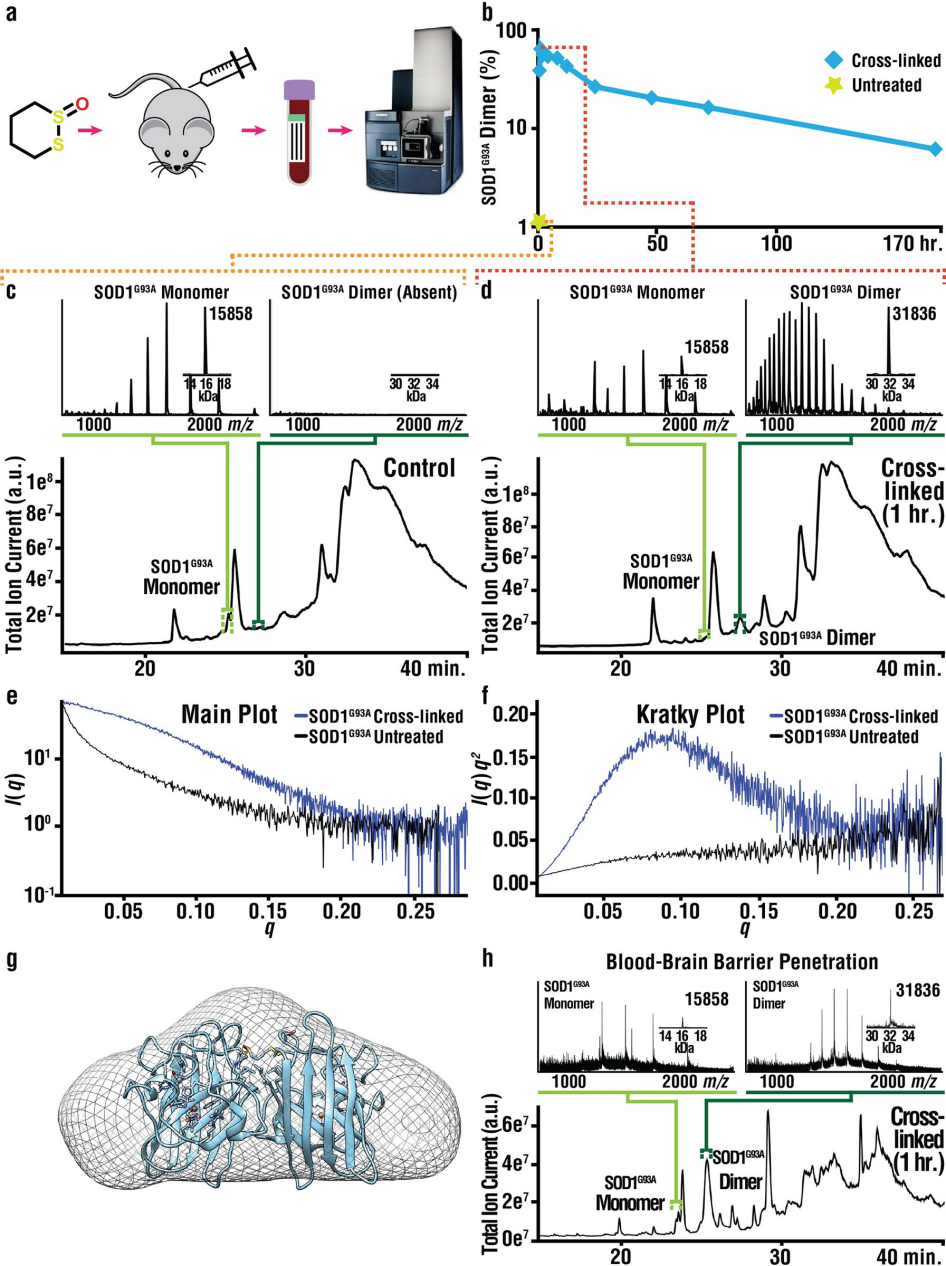
*S*-XL6 cross-links SOD1 in an ALS mouse model and reduces aggregation in vitro. **(A)** Schematic of the pharmacodynamic workflow. **(B)** Pharmacodynamic profiling of RBC proteins by LC-MS analysis indicates the formation of an *S*-XL6 cross-linked dimer in treated SOD1^G93A^ mice. Hemizygous fALS SOD1^G93A^ mice were dosed once at 10 mg/kg with *S*-XL6 via tail vein injection, blood was collected periodically over a 7-day period, and LC-MS analysis **(C and D)** was performed to assess percentage of cross-linked SOD1 (details in **S1 Table)**. Spectra represent a 6-s average at peak apex. (**E and F**) In vitro SAXS experiments for SOD1^G93A^: Semi-log plot of the scattering intensity (I, log scale) as a function of momentum transfer, *q*; and Kratky plot [*q*^*2*^•*I*(*q*) versus *q*]. The plot shape for untreated SOD1^G93A^ is consistent with a heterogenous mixture of unfolded proteins including amorphous aggregates, whereas plot shape for cross-linked SOD1^G93A^ is consistent with a folded, globular structure. The estimated radius of gyration is approximately 20 Å. (**G**) A 3-D reconstruction of cross-linked SOD1^G93A^ is superimposed upon the SOD1^G93A^ crystal structure (PDB: 3GZO). (**H**) Target engagement via LC-MS analysis in transgenic (Tg) SOD1^G93A^ mouse brain dosed once at 30 mg/kg with *S*-XL6 via subcutaneous injection. The data underlying this figure can be found in S1 Data. ALS, amyotrophic lateral sclerosis; fALS, familial amyotrophic lateral sclerosis; LC-MS, liquid chromatography-mass spectrometry; RBC, red blood cell; SOD1, superoxide dismutase 1.

Previous studies have shown that SAXS can monitor changes to protein tertiary and quaternary structure in solution. Therefore, SAXS (**Fig 9E and 9F**) was performed to determine the effects of cross-linking upon the structure of fALS variant SOD1^G93A^, the same variant expressed by the mice that were dosed in the present study. Whereas previous SAXS studies of SOD1^G93A^ preparations showed a radius of gyration (*R*g) of 20 to 24 Å [14,47], our SAXS data for as-isolated and untreated SOD1^G93A^ could not be fit to determine an *R*g, probably due to the aggregation of SOD1. The *R*g of 20 Å estimated from scattering data was consistent with *S*-XL6 treated SOD1^G93A^ being a native dimer.

### Pharmacodynamic profiling, stabilization of SOD1^G93A^ in vivo, and blood–brain barrier penetration

To assess the time course of *S*-XL6 target engagement in vivo, pharmacodynamic (PD) profiling was carried out using B6 G93A SOD1 mice [13]. Mice were dosed with *S*-XL6 via intravenous (IV) injection and blood was collected at different time points, post dose, from the tail vein. To detect the intact SOD1^G93A^ protein-cross-linker complex, we utilized a facile MS assay, namely a combination of solvent-extraction, hemoglobin precipitation, and liquid chromatography coupled with mass spectrometry (LC-MS) [48]. We observed that a single IV dose of *S*-XL6 at 10 mg/kg converted 63% of the SOD1^G93A^ (**Fig 9D**) into a cross-linked dimer in blood at 1-h post-dose by the intended MOA (**Fig 1**), whereas untreated mice showed only SOD1 monomer (**Fig 9C**). A half-life improvement was observed for cross-linked SOD1^G93A^ (**Fig 9B**, 26 h 1-compartment model, 11 and 68 h using a 2-compartment model) compared to the 10-h half-life reported for untreated SOD1^G93A^ in vivo [49]. Next, we assessed *S*-XL6’s target engagement in SOD1^G93A^ mouse brain (**Fig 9H**). Dosed (30 mg/kg via SC) mice showed 86% conversion of SOD1 to cross-linked dimer at 1 h.

### Survival studies

**Fig 10:**
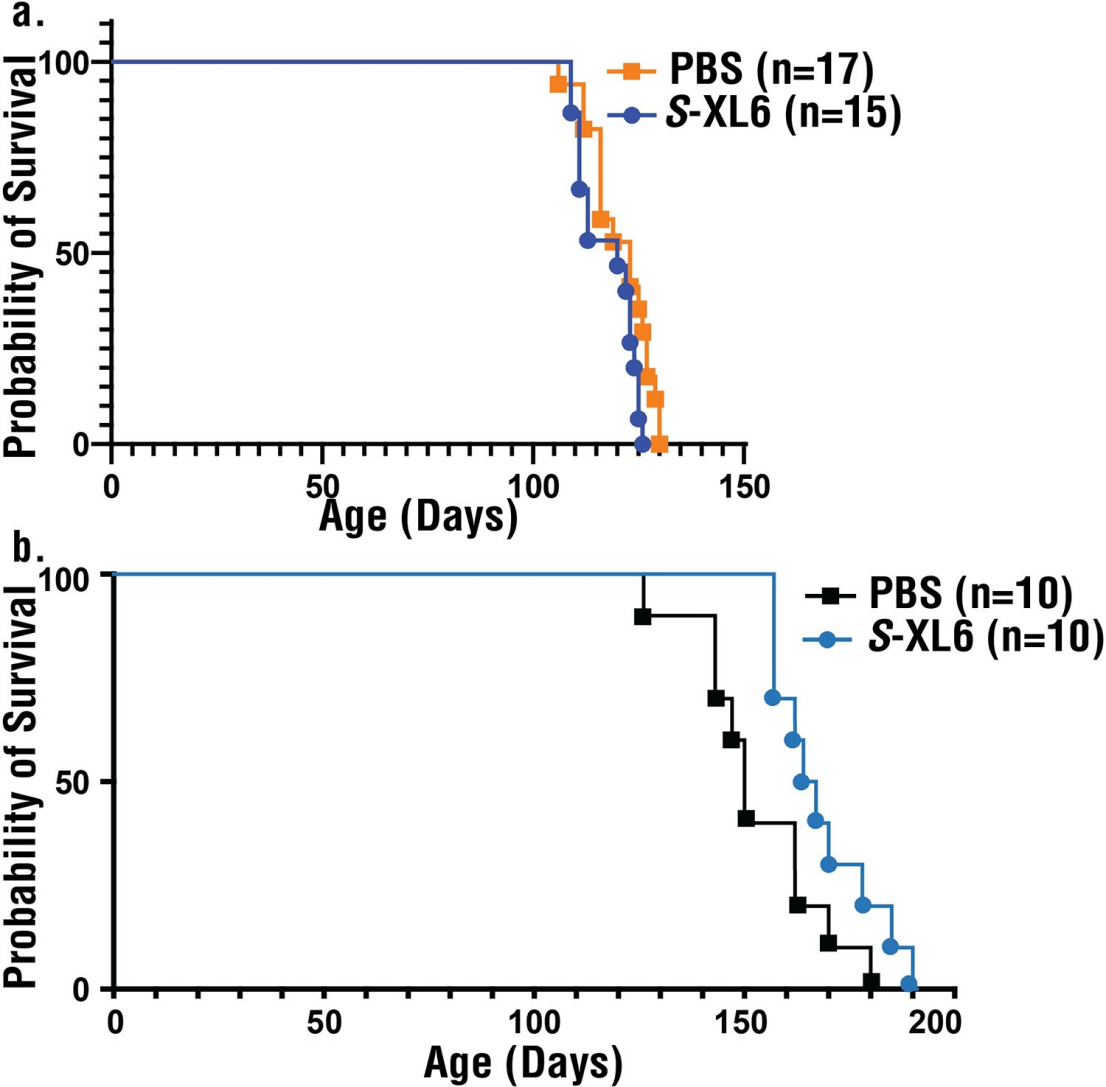
Survival studies of fALS mice treated with subcutaneously administered S-XL6. **(a)** Treatment with *S*-XL6 in B6SJL G93A (hybrid line) mice began at age 60 days in cohorts of 40 mice (18 untreated and 18 treated until end-stage; 2 mice from each group dosed for *ca*. 50 days before being killed for target engagement assessment in brain). After 1 week at a 20 mg/kg dose target engagement in blood was monitored as shown in **Fig 9**. Based upon these results, to improve target engagement to >50% peak effect, the dose was increased to 50 mg/kg. No survival improvement was observed in B6SJL mice. Body weight, disease score, and all paws’ grip score analysis for B6SJL mice are given in **S8 Fig. (b)** B6 G93A/YFP (congenic line) mice were dosed on weekdays starting from day 108 to endpoint and demonstrated a modest survival improvement. Immediately after dosing mice exhibited lethargy and shaking that subsided after 20 min. The data underlying this figure can be found in S1 Data. fALS, familial amyotrophic lateral sclerosis.

Two fALS lines were used to assess survival in genetic backgrounds with different disease severity. The hybrid “B6SJL G93A mice” are commonly used for fALS SOD1 survival studies, exhibit earlier onset (approximately 125 days), have a mixed B6/SJL background, and are generally less susceptible to environmental stress. A second and congenic line was obtained by crossing (>10 generation) fALS “B6 G93A mice” with “YFP-16 mice” [50], expresses YFP in motor and sensory neurons to enable further methods development for target engagement profiling at cellular-resolution [51], exhibit later onset (approximately 155 days), and has a pure C57BL/6J genetic background. Dose and dose regimen were estimated from the blood PK/PD profile and BBB penetration described above. A subcutaneous (SC) route of administration was chosen because in our hands it results in more precise delivery. After an initial (SC) dose of 20 mg/kg B6SJL G93A mice, the PK/PD profile demonstrated lower target engagement than expected (**Figs 10 and S8**) and the dose was increased to 50 mg/kg (dosed once a day, Monday to Friday) from day 70 onward. This dose provided no survival benefit in B6SJL G93A mice. The same dose, starting from day 108, provided a modest survival benefit in B6 G93A mice (153 +/− 15 days, control; 169 +/− 11 (STD) days dosed). In both studies, mice were gender and littermate matched and non ALS-related deaths were excluded. We are currently developing the methods necessary for quantifying copy number and cannot rule out reduced SOD1 expression as having contributed to the survival benefit in treated B6 G93A mice. Given this and that the B6SJL G93A results were obtained in a standard genetic background by an experienced laboratory (Brown) and the B6 G93A results were obtained in a novel line (with YFP expressed in motor neurons) by a less experience laboratory (Agar), we interpret these survival results conservatively as there having been modest to no overall benefit (or toxicity) from *S*-XL6 administration when approximately 40% of SOD1 is engaged (on average) over time. As detailed below, we believe that there is room for considerably improved target engagement in mice, but this will require improved bioanalytical methods for covalent drug PK/PD assessment.

## Discussion

We have shown that a cyclic thiosulfinate cross-linker, *S*-XL6, binds to Cys 111, cross-links monomers, and stabilizes the dimer of SOD1 fALS variants with diverse physicochemical properties and disease severities. Notably, *S*-XL6 has promising preclinical properties, including: oral bioavailability, avoiding plasma proteins, low toxicity (*c*.*a*. 1 g/kg/day LD_50_ via PO), conforming to Lipinski’s rules, and exhibiting BBB penetration. Notably, treatment with *S*-XL6 extends the biological half-life of SOD1^G93A^ [49]. The large degree of stabilization of fALS variants by *S*-XL6 is promising. First, loss of thermodynamic stability is the largest epidemiological risk factor for fALS. Second, loss of thermodynamic stability (hazard ratio = 24) and gain of aggregation propensity (hazard ratio = 13) act synergistically (combined hazard = 333), indicating that one has an effect upon the other [9,52]. This is expected given that protein misfolding and decreased thermodynamic stability generally increase the rate for the nucleation phase of aggregation. S-XL6 promoted the aggregation of EGFP-conjugated wild-type SOD1, but not SOD1^G93A^, in the NSC-34 cell line, and had no effect on the survival. Our results indicate that S-XL6 treatment provides modest (congenic G93A line) to no (hybrid G93A line) survival benefit in fALS SOD1 mouse models. We believe that further studies are necessary to fully assess the therapeutic potential of cyclic thiosulfinate-mediated fALS SOD1 stabilization (and kinetic stabilization of SOD1). First, the pharmacological burden of stabilizing SOD1 in a mouse model is much higher than in humans, because mice express 28 copies of G93A SOD1 and presumably require 14 times higher dose than humans. Second, fALS SOD1 patients (other than D90A homozygotes) express a fALS SOD1 variant and wild-type SOD1, which are believed to interact. Finally, the bioanalytical methods and mathematical models required to assess and optimize target engagement of covalent drugs (and S-XL6) in vivo are currently insufficient. This is the result of the established uncoupling of the pharmacokinetics and pharmacodynamics (e.g., lack of correlation between free drug concentration and effect) for drugs that do not reversibly bind their targets. We are currently developing the necessary analytical techniques and mathematical models to fill this need and are addressing the safety and selectivity profile of *S*-XL6. Despite its limitations, this study provides important benchmarks for further compound development, and the first chemical tool capable of testing the therapeutic hypothesis that kinetic stabilization of the fALS SOD1 dimer stabilization will improve survival. The most effective preclinical therapeutic strategy for SOD1-related fALS has been the stabilization of SOD1 via the CuATSM (Cu (II)-diacetyl-bis(N(4)-methylthiosemicarbazone))-mediated incorporation of metals [53]. Given that *S*-XL6 improves metal binding through stabilization, and CuATSM delivers metals, we anticipate a benefit to combining these approaches.

**Table 1:** In vitro and in vivo properties of *S*-XL6. This table summarizes the results from our preclinical development studies of *S*-XL6.

Characteristics	Result
Purity	99% pure by NMR
Melting point (°C)	81–85
Cytotoxicity LC_50_ (μm)	446
EC_50_ (μm)	~5
Mechanism of action (MoA)	Cross-links SOD1 monomers via Cys111 residues
Maximum solubility (mM)	360 (pH 7.4)
Impact of cross-linking on SOD1 structure (SAXS and HDX-MS)	Cross-linked quaternary structures of fALS SOD1 variants are more comparable to wild-type SOD1
Impact of cross-linking on the thermal stability of SOD1 ΔTu (°C)	*Wild-type*: +14, A4V: +16, G93A: +14, H46R: +23, G85R: +24
Enzymatic activity	Activity of cross-linked quaternary structures of fALS SOD1 variants are more comparable to uncross-linked *wild-type* SOD1
Acute toxicity (Mouse) LD_50_ (mg/kg)	Per oral (PO): 1,000; IV: 75; SC: 275
Target engagement in blood and brain	SOD1 dimer formation in G93A fALS mouse blood and brain
log BB (% BBB penetration)	0.02 (105%)
Target engagement (mouse) *t*_*1/2*_ *S*-XL6 + SOD1 (h)	26 h (1-compartment model) 11 and 68 h (2-compartment model)

fALS, familial amyotrophic lateral sclerosis; IV, intravenous; SC, subcutaneous; SOD1, superoxide dismutase 1.

## Materials and methods

### Ethics statement

Work with vertebrate animals were conducted at Northeastern University and UMass Chan Medical School and were performed according to the Guide for the Care and Use of Laboratory Animals (National Institutes of Health, Bethesda, Maryland, United States of America). All methods and animal manipulations performed by the Agar laboratory were approved by the Northeastern University Institutional Animal Care and Use Committee (IACUC) under protocol #16-0303-R and manipulations performed by the Brown laboratory were approved by the University of Massachusetts Chan Medical Institutional Animal Care and Use Committee under protocol #201900287. No human experiments were performed.

### Synthesis of cyclic thiosulfinate 1,2-dithiane-1-oxide (*S*-XL6)

**Fig 11:**
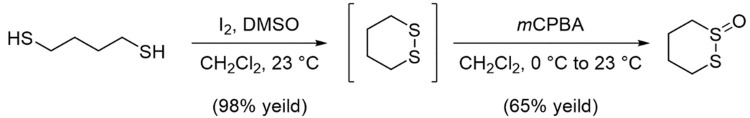
Synthesis of cyclic thiosulfinate 1,2-dithiane-1-oxide (*S*-XL6).

Synthesis of 1,2-dithiane-1-oxide (*S*-XL6) was achieved according to the previously published literature procedure [28]. To a round-bottom flask was added I_2_ (2.08 g, 8.18 mmol, 0.20 equiv.) and DMSO (2.91 mL, 40.90 mmol, 1.0 equiv.). The resulting dark brown solution was stirred gently. 1,4-butanedithiol (4.76 mL, 40.90 mmol, 1.0 equiv.) was dissolved in CH_2_Cl_2_ (16.3 mL) and added dropwise to the stirring solution of I_2_ in DMSO (**Fig 11**). The rate of stirring was increased gradually with the addition of 1,4-butanedithiol in CH_2_Cl_2_. Upon completion of addition the resulting solution was stirred for 1 h at 23°C. The light brown solution was quenched with slow addition of 10% aq. Na_2_S_2_O_3_ (25 mL). The layers were separated, and the aqueous layer was extracted with CH_2_Cl_2_ (3 × 50 mL). The combined organic layers were concentrated under reduced pressure. The concentrated light-yellow oil was taken up in 100 mL EtOAc and washed with sat. aq. NaCl (50 mL). The layers were separated, and the aqueous layer was extracted with EtOAc (3 × 75 mL). The combined organic layers were dried over Na_2_SO_4_ and concentrated under reduced pressure to give pure 1,2-dithiane (4.82 g, 98% yield) as a faint yellow solid. Rf = 0.80 (5:1 hexanes:EtOAc). 1,2-dithiane was then used without further purification to form 1,2-dithiane-1-oxide (*S*-XL6). In a round-bottom flask, 1,2-dithiane (4.82 g, 40.09 mmol, 1.0 equiv.) was dissolved in CH_2_Cl_2_ (40.0 mL) and cooled to 0°C. A solution of *m*CPBA (9.48 g, 73% weight, 40.09 mmol, 1.0 equiv) in CH_2_Cl_2_ (100.0 mL) was added dropwise via addition funnel. The resulting colorless, cloudy solution was allowed to stir at 0°C for 1 h while warming to 23°C and allowed to stir 1 h at 23°C. The reaction was cooled again to 0°C and quenched with solid Na_2_CO_3_ (40.0 g, 377 mmol, 1 g per mmol *m*CPBA). The resulting dense slurry was filtered through a short plug of celite and concentrated under reduced pressure. The resulting cloudy oil was purified by flash column chromatography on silica gel with 2% MeOH/CH_2_Cl_2_ to give *S*-XL6 (3.53 g, 64% yield) as a colorless solid. Rf = 0.33 (2% MeOH / CH_2_Cl_2_). ^1^H NMR (500 MHz, CDCl_3_, δ): 1.82–1.91 (m, 1H), 1.95–2.08 (dtt, J = 14.0, 12.8, 3.0 Hz, 1H), 2.09–2.17 (m, 1H), 2.60–2.73 (m, 2H), 3.03–3.13 (dt, J = 13.2, 3.0 Hz, 1H), 3.17–3.24 (td, J = 13.4, 3.7 Hz, 1H), 3.61–3.71 (ddd, J = 14.0, 12.0, 2.5 Hz, 1H) (**S4 Fig**); ^13^C NMR (100 MHz, CDCl_3_, δ): 15.29, 23.47, 25.74, 51.92 (**S5 Fig**). [M+H]^+^ was observed for C_4_H_8_OS_2_ and found 137.00914 Da (**S6 Fig)** via Bruker 9.4T SolariX XR Mass Spectrometer (Bruker, Billerica, Massachusetts, USA). Melting point found to be 83–86°C (lit 83–86°C).

### Expression and purification of *wild-type* SOD1, SOD1^A4V^, SOD1^G93A^, SOD1^H46R^, and SOD1^G85R^

Expression and purification of SOD1 were conducted as previously published [22]. Briefly, EGy118ΔSOD1 yeast were transformed with a *wild-type* SOD1, SOD1^A4V^, SOD1^G93A^, SOD1^H46R^, or SOD1^G85R^ YEp351 expression vector and grown at 30°C for 44 h. Cultures were centrifuged, lysed in a blender using 0.5 mm glass beads, and subjected to a 60% ammonium sulfate precipitation. Then, the sample was centrifuged, and the resulting supernatant was diluted to 2.0 M ammonium sulfate. The diluted sample was passed through a phenyl-sepharose 6 fast flow (high sub) hydrophobic interaction chromatography column (Cytiva Life Sciences, Marlborough, Massachusetts, USA) using a linearly decreasing salt gradient from high salt buffer (2.0 M ammonium sulfate, 50 mM potassium phosphate dibasic, 150 mM sodium chloride, 0.1 mM EDTA, 0.25 mM DTT (pH 7.0)) to low salt buffer (50 mM potassium phosphate dibasic, 150 mM sodium chloride, 0.1 mM EDTA, 0.25 mM DTT (pH 7.0)) over 300 mL. Fractions containing SOD1 eluted between 1.6 and 1.1 M ammonium sulfate and were confirmed with SDS-PAGE. These fractions were pooled and exchanged into low salt buffer (10 mM Tris (pH 8.0)). Pooled fractions were then passed through a Mono Q 10/100 anion exchange column (Cytiva Life Sciences, Marlborough, Massachusetts, USA) using a linearly increasing salt gradient from low salt buffer to high salt buffer (10 mM Tris (pH 8.0), 1 M sodium chloride) from 0% to 30%. SOD1 fractions were collected between 5% and 12% high salt buffer and were confirmed with SDS-PAGE, western blot, and Fourier-transform ion cyclotron resonance mass spectrometry (FT-ICR-MS).

### Confirmation of cross-link formation in vitro

*Wild-type* SOD1, SOD1^A4V^, SOD1^G93A^, SOD1^H46R^, and SOD1^G85R^ stock solutions were diluted to 40 μm in 10 mM ammonium acetate (pH 7.4). DMSO stock of *S*-XL6 was used to prepare 400 μm (10×) in 10 mM ammonium acetate (0.5% DMSO). Protein and cross-linker samples were combined in equal volumes (final concentration 20 μm SOD1, 200 μm cross-linkers, 0.25% DMSO) and incubated at 37°C for 4 h at 350 rpm. Complete cross-linking was confirmed by mass spectrometry on a 9.4T Bruker SolariX (Bruker Corporation, Billerica, Massachusetts) via direct infusion as previously described [54,55]. Prior to infusion, samples were diluted to 1 μm in 50:50 acetonitrile:water, with 0.1% formic acid. During analysis, 32 scans were acquired in positive mode and averaged. Funnel 1 and skimmer 1 were kept around 150 V and 20 V, respectively, and funnel RF amplitude was held at 60.0 Vpp.

### Differential scanning fluorimetry

The effect of cross-link formation on the thermal stability of SOD1 was determined by differential scanning fluorimetry as previously described [22]. *Wild-type* SOD1, SOD1^A4V^, SOD1^G93A^, SOD1^H46R^, and SOD1^G85R^ (20 μm in 10 mM ammonium acetate (pH 7.4)) were incubated with 10-fold molar excess *S*-XL6 for 4 h at 37°C in protein low bind Eppendorf tubes using Eppendorf Thermomixer at 350 rpm (Eppendorf North America, Enfield, Connecticut, USA). After incubation, SYPRO Orange (Invitrogen Corporation, Carlsbad, California, USA), an environmentally sensitive fluorescent dye which is quenched in an aqueous environment but becomes unquenched once it binds hydrophobic residues, was added to the reaction mixture to a final concentration of 200×. The samples were then transferred to a fast optical 96-well reaction plate (Applied Biosystem, Life Technologies Corporation, Carlsbad, California, USA) and loaded on to the real-time PCR machine (Applied Biosystem, Life Technologies Corporation, Carlsbad, California, USA) after plate was spun down to eliminate bubbles. The Melt curve template in StepOne Plus software was used to set up a method. The SYBR reporter and ROX quencher data were collected for 35 cycles with a temperature gradient ranging from 25 to 99.9°C at a ramp rate of 0.5°C/min. The final concentration of DMSO in the reaction mixture was checked as it contributes to the denaturation of proteins. Dilutions were performed accordingly to maintain the final concentration of DMSO less than 1% (vol/vol). All samples were run in technical triplicates. The background data was subtracted from the average relative fluorescence and average derivate for each temperature point, and the data were normalized before T_u_s were quantified by averaging the negative first derivative of relative fluorescence of all 3 runs and identifying the local minima.

### Crystallographic structure determination

Crystals of SOD1 grown by combination of hanging drop method with micro seeding. For crystallization 2 μl of 10 mg/ml of recombinant *wild-type* SOD1 were mixed with 2 μl of crystallization solution containing 2.5 M ammonium sulfate 150 mM NaCl, 50mM acetate buffer (pH 4.75) and equilibrated over 400 μl well containing the same crystallization solution. To make *S*-XL6 complex, crystals that appear as plates were soaked for 2 h in stabilizing solution (3 M ammonium sulfate 150 mM NaCl Acetate buffer (pH 4.75)) mixed with DMSO stock of 50 mM *S*-XL6 in 1:16 ratio, to final concentration of *S*-XL6 3 mM. For the as-isolated *wild-type* SOD1 Paratone-N oil was used as a cryo protectant. X-ray data was collected using the FRE+ X-ray source coupled to an EIGER R 4M photon counting detector at 1.54 Å wavelength to 1.67 Å resolution for *S*-XL6 complex and to 1.77 Å resolution for as-isolated *wild-type* SOD1. Data were processed with Mosflm [56]. Structures were solved by molecular replacement with Molrep [57] using starting model 2C9V with following isotropic refinement in CCP4i2 with refmac5 [58]. The cycles of refinement have been iterated with model rebuilding in COOT [59]. Final structures have been validated with Molprobity [60] and submitted to PDB ID with 8CCX for *S*-XL6 complex and 8Q6M for as-isolated *wild-type* SOD1, used as control. Data collection and refinement statistics are summarized in S2 Table.

### Hep G2 cell culture

The Hep G2 human hepatocarcinoma cell line (American Type Culture Collection, ATCC, Manassas, Virginia, USA) was cultured using EMEM (Eagle’s minimal essential medium, ATCC) supplemented with 10% FBS (fetal bovine serum, ATCC) and 1% penicillin streptomycin as previously described [45] (10,000 units/mL penicillin and 10,000 μg/mL streptomycin, Fisher scientific, Hampton, New Hampshire, USA). The cells were grown and subcultured around 72 h at 37°C, 5% CO_2_ under controlled humidity. The cells growth and morphology were inspected using an inverted Carl Zeiss microscope (Carl Zeiss Microscopy LLC, White Plains, New York, USA).

### Cell viability assay/cell cytotoxicity assay

Using manufacturer’s instructions, Hep G2 cells were cultured for 24 h with a density of 2 × 10^5^ cells/mL in a 96-well culture plate at 37°C, 5% CO_2_ under controlled humidity. The cells were treated with *S*-XL6 ranging with 10, 100, 150, 250, 400, 600, 800, and 1,000 μm for 24 h (0.1% DMSO). Cell cytotoxicity was performed via the MTT (3-(4,5-dimethylthiazol-2-yl)-2,5-diphenyltetrazolium bromide) assay at 570 nm absorbance using BioTek synergy H1 (Vermont, USA) plate reader according to previously published paper [45]. The MTT stock was made at 5 mg/mL using 0.9% sodium chloride solution which was purchased from Sigma Aldrich (St. Louis, Missouri, USA). Twenty-four hours after dosing the plate, the cultured medium was aspirated and replaced with the equal volume of MTT solution (10× dilution using EMEM complete media). The plate was incubated for 4 h (37°C, 5% CO_2_). The MTT solution was replaced by the equal amount of acidified isopropanol (0.3% hydrochloric acid (v/v)) and then formed formazan was dissolved by shaking the plate gently for 30 min. The absorbance was measured at 570 nm for both the samples and acidified isopropanol for background calculation. Chlorpromazine and cells with 0.1% DMSO were used as positive control and vehicle, respectively. The cell viability of each concentration was measured by normalizing against mean value of cells with 0.1% DMSO (vehicle).

### SDS-PAGE and western blot

The Hep G2 human hepatocarcinoma cell line (American Type Culture Collection, ATCC, Manassas, Virginia, USA) was cultured using complete EMEM (Eagle’s minimal essential medium, ATCC) media including 10% FBS (fetal bovine serum, ATCC) and 1% penicillin streptomycin (10,000 units/mL penicillin and 10,000 μg/mL streptomycin, Fisher Scientific, Hampton, New Hampshire, USA) [28]. Cells were counted and checked for morphology using an inverted Carl Zeiss microscope (Carl Zeiss Microscopy LLC, White Plains, New York, USA). A 96-well plate (Corning Life Sciences, Tewksbury, Massachusetts, USA) was used for culturing cells at 0.2 mill/mL concentration for 24 h at 37°C, 5% CO_2_ under controlled humidity for western blot experiment. Cells were dosed with *S*-XL6 in triplicate (wells) at different concentrations (0.001 μm through 200 μm) and incubated for 30 min by replacing the existing EMEM with PBS buffer. *Wild-type* SOD1 purified from human erythrocyte (Sigma Aldrich, St. Louis, Missouri, USA) was used as positive control (standard) in the experiment. The growth buffer was aspirated and replaced with 30 μL of lysis buffer (150 mM NaCl, 1% Triton X-100, 0.5% sodium deoxycholate, 0.1% SDS, and 50 mM Tris (pH 8.0), all chemicals were purchased from Sigma Aldrich (St. Louis, Missouri, USA)) and incubated for 10 min followed by addition of non-reducing sample buffer. Samples were loaded into a Bio-Rad mini TGX gel (Bio-Rad Life Sciences, Hercules, California, USA) along with PageRuler prestained protein ladder (Thermo Fisher Scientific, Waltham, Massachusetts, USA) and ran using a Bio-Rad electrophoresis cell. The gel was removed carefully and transferred into a beaker filled with transfer buffer (25 mM Tris, 192 mM glycine, 0.1% SDS, and 10 mM β-mercaptoethanol, all chemicals were purchased from Sigma Aldrich (St. Louis, Missouri, USA)) and incubated briefly at 90°C and then transferred to a Trans-Blot Turbo mini nitrocellulose membrane using Trans-Blot Turbo Transfer system (Bio-Rad, Hercules, California, USA). The membrane was incubated overnight in antibody buffer (50 mM Tris-HCl, 150 mM sodium chloride, 0.1% tween-20, 2.5% dry milk, all chemicals were purchased from Sigma Aldrich (St. Louis, Missouri, USA)) with Cu/Zn SOD polyclonal antibody (1:1,000 dilution, Enzo Life Sciences, Farmingdale, New York, USA). The membrane was then incubated with HRP-linked antibody buffer (1:1,000 dilution, Cell Signaling Technology, Danvers, Massachusetts, USA). The membrane was incubated briefly in the dark with enhanced chemiluminescent (ECL, Thermo Fisher Scientific, Waltham, Massachusetts, USA), followed by imaging using BioRad Chemidoc MP Imaging System (Bio-Rad, Hercules, Massachusetts, USA).

### Gel-based SOD1 enzymatic activity assay

Enzymatic activity of SOD1 variants were assessed using a gel-based assay with riboflavin and nitroblue tetrazolium (NBT) as previously described [61,62]. *Wild-type* SOD1, SOD1^A4V^, SOD1^G93A^, SOD1^H46R^, and SOD1^G85R^ were incubated with or without 10-fold molar excess *S*-XL6 at 37°C for 4 h at 350 rpm in 10 mM ammonium acetate (pH 7.4) (DMSO content <1%). The samples were mixed with native sample buffer (Bio-Rad) in a 1:1 ratio and were loaded into each well to a 12% polyacrylamide gel (Bio-Rad). The gel was stained in dark using 50 mM potassium phosphate (pH 7.8), 0.13 mg/mL of NBT, and 0.1 mg/mL of riboflavin for 45 min. The gel was exposed to light for with 1 μL/mL of *N*,*N*,*N*^′^,*N*^′^-tetramethylethylenediamine (TEMED) for 2 min. SOD1 activity can be seen as colorless bands as the SOD1 scavenges the superoxide that inhibits insoluble blue color formazan formation.

### Chemical-based SOD1 enzymatic activity assay

All samples were prepared as described in gel-based enzymatic activity assay section and were (10 μL) mixed with 160 μL of solution A (6,000 units/mL catalase, 10 mM hypoxanthine, and 5 mM XTT) and 160 μL of solution B (xanthine oxidase 16 mg/mL) [61] in a flat-bottom 96-well plate and was monitored for absorbance at 490 nm for 30 min. All samples were analyzed in technical triplicate using slope function in Microsoft Excel.

### Size exclusion chromatography (SEC)

We have analyzed samples with and without cross-linker via SEC with high performance liquid chromatography (SEC-HPLC) to assess the SOD1 protein composition in its native state [63,64]. Wild-type SOD1, SOD1^G93A^, SOD1^A4V^, SOD1^H46R^, and SOD1^G85R^ (20 μm stock) were incubated with or without 10-fold molar excess *S*-XL6 in 200 mM ammonium acetate (pH 7.4) or 4 h at 350 rpm, and each cross-linked dimer were tested using a Bruker 9.4T FT-ICRMS mass spectrometry. For inducing aggregation, all samples (final conc. 1 μm) were incubated at 37°C for 1 h prior the SEC analysis. An Agilent HPLC 1290 Infinity II LC system was used for duplicate analysis of each sample type by injecting 5 μL sample via Waters Acquity protein BEH SEC column (125 Å, 1.7 μm, 4.6 mm × 150 mm, Waters Corporation, Milford, Massachusetts, USA). An isocratic mobile phase consisting of 100 mM sodium phosphate, 200 mM sodium chloride (pH 7.4) was used for 6 min to analyze each sample at wavelength of 280 nm. Waters BEH 200 standard was injected at the beginning, middle, and at the end to confirm the retention time was not shifting throughout the entire experiment.

### NSC-34 cell culturing and transfection

Mouse neuroblastoma/motor neuron hybrid cells (NSC-34) [65] were cultured and maintained as previously described [42]. Briefly, NSC-34 cells were cultured in Dulbecco’s Modified Eagle’s Medium with F12 supplement (DMEM/F12, Invitrogen Australia) containing 10% (v/v) heat-inactivated fetal bovine serum (FBS, Bovogen Biologicals, Australia) and 2 mM L-Glutamine (Invitrogen, Australia). Cells were passaged using 0.25% trypsin, 0.02% EDTA (Invitrogen, Australia). Cells were maintained within humidified incubators at 37°C with 5% CO_2_.

To transfect NSC-34 cells, they were first plated out to 40% confluency in either 6-well or 12-well tissue culture-treated plates and cultured for 24 h. Following this, TransIT-X2 reagent (Mirus Bio, USA) was used according to the manufacturer’s instructions to transfect NSC-34’s (1 μg DNA per well for 12-well plates, 2.5 μg per well for a 6-well plate). Cells were then incubated for 8 h prior to replating for experiments into other plates or chambers.

### Immunoblotting for SOD1-EGFP

Transfected NSC-34 cells were plated out into 12-well plates and left overnight. The following day, cells were treated with *S*-XL6 (20 μm) or vehicle (0.04% v/v DMSO) and incubated for another 24 h. Cells were then harvested via aspiration and pelleted (300 × *g*). Lysis and immunoblots were carried out as previously described [33]. Briefly, cell pellets were lysed in ice-cold lysis buffer containing protease inhibitors (Thermo Fisher, USA) and N-ethylmaleimide (NEM) at a final concentration of 1 mg/mL. Protein concentration was measured by DC assay (BioRad, Australia) and samples were denatured in SDS-sample buffer containing 40 mM NEM at 90°C for 5 min. Lysates were loaded into 26-lane stain-free gels (BioRad, Australia) and electrophoresed. Proteins were transferred to 0.2 μm PVDF membranes for 1 h at 4°C. Membranes were blocked in 5% skim milk in TBST for 1 h prior to addition to primary antibody which as a rabbit anti-GFP antibody (ab290, 1:5,000, Abcam USA) and incubated overnight at 4°C. Membranes were washed 3× and then incubated for 1 h at room temperature in secondary antibody (goat anti-rabbit HRP conjugate at 1:5,000 (catalog no.: P0448; Dako). Membranes were then washed 3× prior to developing with SuperSignal West Pico Plus substrate (Thermo Fisher Scientific).

### Determination of cells containing inclusions

Transfected NSC-34 cells were plated out into 96-well plates and left overnight. The following day, cells were treated with various concentrations of *S*-XL6 (1.25–20 μm) or vehicle (0.0025% to 0.04% v/v DMSO) and incubated for another 48 h. Cells were then fixed using 4% (w/v) paraformaldehyde in 1×PBS for 20 min. Plates for imaging were then processed as previously described [33] with some changes. Cells were imaged using a DMi8 epifluorescent microscope (Leica, Germany) fitted with a Mercury Arc Lamp, a 10× 0.25NA (CAT NO) air objective and an automated stage [33]. A software-based autofocus strategy was utilized to focus on the GFP signal within cells, with a subsequent image being taken in the DAPI channel to view Hoescht-stained nuclei. Relative focus correction was applied between channels to offset emission wavelength effects to the focal plane. Following image acquisition, images were corrected for illumination variation via a background method and processed using a combination of FIJI [66], StarDist, CellProfiler [66], and CellProfiler Analyst [67] as previously described [33].

### Determination of relative cell viability and relative SOD1-EGFP levels

Image set data taken from the previous inclusion formation assay were used to examine both relative cell viability and relative levels of SOD1-EGFP. To determine relative viability, we extracted the total number of transfected cells in each treatment group and normalized the values to the untreated group to determine the relative number of cells at assay endpoint.

For EGFP level analysis, part of the data analysis used to determine which cells have or do not have inclusions is the measurement of mean cell fluorescence intensity in the GFP channel. This value was extracted from the imaging data and plotted as a mean per drug treatment.

### Mouse dosing and SOD1 isolation

Pharmacodynamic profiling was carried out using hemizygous mice expressing human SOD1^G93A^ (“fast-line” Jackson Laboratory; B6SJL-Tg(SOD1*G93A)1Gur/J, also known as SOD1-G93A stock- 002726) [13]. Mice were bred to express YFP in neurons in anticipation of BBB-penetration assays and Matrix-Assisted Laser Desorption Ionization Mass Spectrometry Imaging (MALDI MSI) analysis according to the published protocol [68,69]. Extraction of SOD1^G93A^ from red blood cell (RBC) protein was performed as previously described [48]. *S*-XL6 was prepared in 1× PBS and mice were dosed at 10 mg/kg via IV injection in their lateral tail vein slowly and gently. Mice were trapped in restrainer and around 40 μL of blood was collected in Greiner Bio-one K_3_EDTA tubes (Greiner Bio-one, North Carolina, USA) post injection at 30 min, 1 h, 2 h, 4 h, 8 h, 12 h, 24 h, 48 h, 72 h, and 168 h. Tubes were centrifuged at 2,000 rpm at 4°C for 5 min immediately after collecting blood. Carefully plasma was discarded and acid citrate dextrose solution (0.48% citric acid, 1.32% sodium citrate, 1.47% glucose, all chemicals were purchased from Sigma Aldrich (St. Louis, Missouri, USA)) was used to wash the sample by centrifuging at 2,000 rpm at 4°C for 5 min. Following wash, the supernatant was removed, and RBC were lysed by the addition of 8× equivalent of 10 mM ammonium acetate. To the hemolysate, 0.15 equivalents of cold chloroform and 0.25 equivalents of cold ethanol was added. Samples were vortexed at 1,800 rpm at 4°C for 15 min and centrifuged at 12,000 rpm for 10 min. The supernatant was collected and stored at −80°C after flash freezing for LC-MS analysis. Prior to LC-MS analysis, samples were acidified to 10% formic acid.

### Confirmation of *S*-XL6 cross-linked SOD1^G93A^ dimer formation in vivo

In vivo cross-linking from purified RBC was confirmed using an H-Class Acquity UPLC (Ultra Performance Liquid Chromatography) system coupled to a Xevo G2-S Q-ToF (Quadrupole Time of Flight) mass spectrometer (Waters Corp, Milford, Massachusetts, USA) as previously described [55,70]. The LC system was equipped with reversed phase Acquity UPLC Protein BEH C4 (300 Å pore size, 1.7 μm particle size, 100 mm bed length, 2.1 mm ID × 100 mm) column at 60°C with a flow rate of 0.2 mL/min. The mobile phase consisted of a mixture of 0.1% formic acid in water (solvent A) and 0.1% formic acid in acetonitrile (solvent B). The sample was introduced in 10% formic acid and 5 μL was injected for analysis. UNIFI software (Waters Corp, Milford, Massachusetts, USA) was used for system control and data processing. Solvents A and B were combined in a gradient: 0 to 2 min: 95% A; 2 to 70 min: 30% A; 72 to 75 min: 5% A; 78 to 80 min: return to initial conditions. The MS was operated in positive mode and calibrated prior to analysis (*ca*. daily). The chromatographic window containing the SOD1^G93A^ monomer and dimer were assigned using EICs (Extracted Ion Chromatogram) *m/z* 1322.5 to 1323.5 (monomer) and *m/z* 1180.0 to 1180.5 (dimer) and mass spectra from this region were summed. Raw MS data from this composite spectrum were deconvoluted and average masses were calculated using the MaxEnt1 algorithm. The ratio of dimer intensity (31836 Da, SD 1.3 Da) to the sum of monomer (15858 Da, SD 0.8 Da) plus dimer intensity were then used to calculate the percentage of SOD1^G93A^ dimer at different time points, and 10 picomole of the SOD1^G93A^ monomer and dimer were analyzed (as above) in individual experiments and their MS signal intensities were within 10% (i.e., the differences in their chromatographic retention and ionization efficiency were within experimental error). The half-life of the *S*-XL6 cross-linked complex was calculated from apparent half-life of disappearance of the % dimer by linear regression of the terminal beta phase (24 to 168 h time point).

### Target engagement in SOD1^G93A^ transgenic mouse brain

*Wild-type* (non-transgenic) C57BL/6 and transgenic (Tg) SOD1^G93A^ ALS mouse were used to determine the SOD1 target engagement in brain using western blot assay. Non-transgenic mouse was used as a negative control, transgenic control mouse received 1× PBS only via SC, and the transgenic-treated mouse received 30 mg/kg of *S*-XL6 via SC; 1 h post-dose the brain was collected by sacrificing the mice. The brain was then homogenized, and sample was prepared in a similar technique as described in the earlier section of pharmacodynamic sample preparation of this manuscript prior LC-MS analysis.

### Determination of acute toxicity (LD_50_) in vivo

The acute toxicity study was performed using C57BL/6 (Jackson Laboratories, Maine) via PO, IV, and SC to determine the LD_50_ (**Table 1 and S7 Fig)** of *S*-XL6 according to the Bruce’s up/down method [71]. The control group (*n* = 3) was dosed with 1× PBS (PO/IV/SC) and 0.25 mg/kg of *S*-XL6 (PO/IV/SC) was the starting dose for the treatment group. Any clinical abnormalities including but not limited to ruffled fur, unstable gait, severe lethargy, and mortality was observed after dosing. The post-dose washout period was maintained for 48 h for both PO, IV, and SC route of administration. For higher PO dosing stock, a suspension of 200 mg/mL of *S*-XL6 was made using a vehicle consisting of 1% (v/v) Tween 80 and 20% (v/v) PEG 400 in sodium citrate buffer (20 mM (pH 5.5)). The final dosage form of *S*-XL6 was made simply by vortexing for 1 min to get uniform micelles prior dosing.

### In vivo study approval

All studies were performed in accordance with the Guide for the Care and Use of Laboratory Animals (National Institutes of Health, Bethesda, Maryland, USA). The protocols for working with mice were approved by the Northeastern University Institutional Animal Care and Use Committee (IACUC) and protocol for working with rats were approved by the Charles River Laboratories IACUC.

### Small-angle x-ray solution scattering (SAXS)

SAXS data were collected at the G1 beamline at Cornell High Energy Synchrotron Source (CHESS). For each sample, 2 measurements were taken, one of the protein with buffer and one with buffer by itself. Solution scattering data were captured every second for 10 frames. The 10 frames of both buffer and protein were then averaged and the buffer was subtracted out to get the scattering for the protein. Samples were run in a 96-well plate and held at 4°C continuously. Data collection was in the scattering angle (*q*) range of 0.008 to 0.71 Å^-1^ and processed using the software, RAW.

### Preparation of SOD1 samples for SAXS analysis

SOD1^G93A^ was prepared at 3 mg/mL (approx. 190 μm) in HEPES buffer (115 mM NaCl, 1.2 mM CaCl_2_, 1.2 mM MgCl_2_, 2.4 mM K_2_HPO_4_, 20 mM HEPES (pH 7.4)). Stock solutions of *S*-XL6 was freshly made at 10 mM in HPLC grade methanol and diluted to 946 μm in HPLC grade water (5-fold concentration of protein); 100 μL of 3 mg/mL protein was mixed with 110 μL of 946 μm of *S*-XL6. Control sample contained 5% MeOH (final conc. 2.5%). Samples were then incubated at 37°C for 6 h to ensure complete cross-linking. After incubation, excess compound was buffer exchanged out of each sample using a 10 kDa MWCO ultrafiltration device, and 320 μL is diluted to 15 mL into HEPES buffer, spun down to approximately 500 μL, and resuspended in another 15 mL of HEPES buffer. After the final spin, samples were removed and concentrated in a smaller ultrafiltration device and brought to approximately 70 μL final volume (4.7 μm). Samples were flash frozen and stored at –80°C prior to analysis.

### SAXS data analysis and reconstruction of molecular envelopes

Programs within the ATSAS suite [72] were used to determine the estimated radius of gyration and three-dimensional molecular envelopes for SOD1^G93A^ with and without *S*-XL6 using the x-ray solution scattering data. The GNOM program was used to evaluate the pair distribution plot using an indirect Fourier transform. SOD1^G93A^ with *S*-XL6 had a *D*_max_ value of approximately 79 Å, whereas untreated SOD1^G93A^ had a much higher *D*_max_ of over 200 Å due to protein unfolding. The GASBOR program was used to generate three-dimensional ab initio models of connected beads to fit the GNOM data, with the number of beads set approximately to the total number of amino acids in the SOD1 constructs. In order to assess the uniqueness of these solutions, 10 bead models were generated without any symmetry applied, then compared and averaged. Figures were produced using CHIMERA [73] followed by superimposition of envelopes.

### Peptide-level hydrogen deuterium exchange mass spectrometry (H/D-X MS)

To 5 μL of 40 μm SOD1 sample (in 10 mM ammonium acetate in H_2_O (pH 7.4)) was added 20 μL of 99% D_2_O sample buffer (10 mM ammonium acetate in D_2_O (pH 7.3)), diluting the concentration of the protein down to 8 μm. For the reference sample, HPLC grade water was added instead of D_2_O buffer. Samples with D_2_O buffer were incubated at 37°C for 5 exposure time points: 15 s, 50 s, 500 s, 1 h, and 4 h. All exchange reactions occurred at 37°C, pH approximately 7.4 to mimic in vivo conditions. The reaction mixture was quenched by the addition of 25 μL of quench buffer (8 M Gunadinium Hydrochloride (GnHCL), 0.5 M tris(2-carboxyethyl) phosphine (TCEP), 0.2 M Citric Acid at pH 2.35), lowering the pH of the final mixture down to pH 2.45, diluting the concentration of protein in the sample down to 4 μm while simultaneously denaturing the protein and reducing the disulfides. For the 15-s time point, to ensure timely quenching of the exchange reaction, D_2_O buffer previously stored at 37°C was added and the incubation itself was performed at room temperature. All samples preparations were performed in technical triplicate, flash frozen immediately after the quenching reaction and stored at −70°C until analysis. Prior to analysis, 50 μL of 0.1% formic acid in H_2_O was added to the sample to reduce the GnHCL concentration down to 2 M, the recommended concentration threshold for the pepsin column. This was immediately followed by injecting the sample onto a Waters UPLC system designed for H/D-X MS analysis where the samples were digested, desalted, and separated, online. The digestion and trapping of peptides was carried out during a 3-min trapping step over an immobilized pepsin column with a flow rate of 100 μL/min in 0.1% formic acid and water at 10°C. The peptides were trapped on an ACQUITY HSS T3 100 Å, 1.8 μm trap column (Waters Corp, Milford, Massachusetts, USA) maintained at 0°C. At the end of the trapping step, within the 0°C chamber, the flow was directed to the ACQUITY HSS T3 100 Å, 1.8 μm analytical column (Waters Corp, Milford, Massachusetts, USA) at 75 μL/min (average back pressure was around 7,500 psi). The analytical separation step was performed over a 9-min gradient of 5% to 25% (0 to 7 min) of buffer B; 25% to 95% (7 to 8.5 min) of buffer B (buffer A, 0.1% formic acid in water, buffer B, 0.1% formic acid in acetonitrile). Eluate from the analytical column was directed into a Waters QToF Synapt G2 HD mass spectrometer with electrospray ionization and lock-mass correction (using the Glu-fibrinogen peptide). Blanks were used between each sample injection to ensure there is no carryover of peptides between runs. Mass spectra were acquired between 50 and 2,000 m/z, in positive polarity and resolution mode. Scan time was set to 0.5 s, cone voltage to 30 V, capillary was 3.5 kV, trap collision energy was 6 V, and desolvation temperature of 175°C.

Prior to H/D-X MS experiments, sample preparation and run conditions were optimized by varying concentrations of GnHCL (2 M, 4 M, 8 M), incubation temperature with D_2_O buffer (4°C, 37°C), flow rate over the pepsin column and HPLC gradient. Conditions listed above yielded the best sequence coverage (greater than 98%) and resolution in the shortest run time. Furthermore, to minimize rate of back exchange from D back to H, the sample mixture pH after the quench reaction was lowered to approximately 2.5 and majority of the run on the instrument was performed at 0°C [74]. All H/D-X MS experiments were performed under identical conditions; therefore, deuterium levels reported are relative and were not corrected for back exchange. Optimized sample preparation and instrument conditions to minimize back exchange from deuterium to hydrogen and technical triplicate measurements for each sample allowed for high confidence comparability assessments between sample types [75].

### H/D-X MS data analysis

Mass spectra and chromatography data were acquired using MassLynx (Waters Corp). The peptides were identified and confirmed via MS^2^ using the PLGS software. The PLGS generated peptide lists and MassLynx acquired mass spectra were imported into the DynamX HDX data analysis software 3.0 (Waters Corp) for further analysis. Only peptides that were identified across all 6 replicates of each SOD1 variant (example: triplicates of untreated *wild-type* SOD1 and *S*-XL6 cross-linked *wild-type* SOD1) were considered. The maximum sequence length of peptides was set to 45, minimum peak intensity set to 10,000, and maximum MH+ error set to 10 ppm. Sequence coverage across all samples achieved was approximately 99% and the average redundancy for covered amino acids was 11. The mass spectra were processed within the DynamX software by centroiding the isotopic distribution of various charge states for all the peptides (typically +2, +3, +4). Deuterium uptake levels were measured by calculating the differences between the centroid of the deuterated peptide versus the undeuterated reference peptide. These mass shifts and differences are plotted against the exchange time points (**S2 Fig** for the SOD1^A4V^, SOD1^H46R^, SOD1^G85R^ N and C terminal peptides). Final percentage uptake was calculated by averaging the percent uptake across technical triplicate measurements utilizing overlapping peptides and recurring residues to elucidate residue level uptake measurements, wherever possible. Three replicates of each sample type were run on different days, and the standard deviation was approximately 3% or less. A conservative threshold of 5% was set during the comparative analysis between sample types and only consistent differences in % deuterium uptake above 5% have been emphasized. Peptic maps were obtained from the DynamX software. Maestro 11.8 (Schrödinger Maestro, New York, USA) was used to map the conformational changes onto the crystal structure of *wild-type* SOD1 (PDB: 1SPD).

### Determination of Cu and Zn metal content

All samples were sent to Element Materials Technology (Santa Fe Springs, California, USA) for quantitation of Cu and Zn metal content via Inductively Coupled Plasma Mass Spectrometry [76]. Briefly, a sample portion (0.05 g) was mixed with internal standards (In-Tb-Sc) and then diluted to a final mass of 5 g with a solution of 0.1% ammonium hydroxide, 0.05% EDTA, and 0.05% Triton X100. The sample appeared to have completely dissolved. Elements were analyzed on an Agilent 7500 ICP-MS (Agilent Technologies) with an octopole collision cell. The standard operating conditions used were RF power: 1,550 W, Sample depth: 8 mm, Carrier gas flow: 1 L/min, Spray chamber temperature: 2°C, Nebulizer Pump: 0.1 rps, Collision gas: 5.4 mL/min helium. For quality control, spike recovery experiments were performed where detection limits of Cu and Zn were measured to be 0.01 ppm and 1 ppm, respectively. Metal content for untreated and *S*-XL6 treated samples are reported in S4 **Table**.

### Proteolytic digestion and MALDI-TOF-MS peptide analysis

Samples of SOD1^A4V^ with or without 10-fold molar excess of *S*-XL6 in 10 mM Tris HCl (pH 7.4) were incubated for 4 h at 37°C [77,78]. After incubation, samples were alkylated with iodoacetamide (100 mM for 30 min), heated to 75°C for 20 min, and then treated with 2 volumes of Poroszyme immobilized trypsin (Applied Biosystems, Life Technologies Corporation, Carlsbad, California, USA) at 37°C for 15 min, mixing every few minutes to keep beads suspended. Beads were removed by centrifugation before analysis. SOD1^H46R^ was incubated with or without 10-fold molar excess of *S*-XL6 and deuterated *S*-XL6 followed by pepsin digestion (1:20 w/w, pepsin:protein) for 120 min. Both the digested samples were analyzed using a microflex MALDI-TOF mass spectrometer (Bruker Daltonics, Billerica, Massachusetts, USA) in reflectron mode in the 2 to 5 kDa range and linear mode in the 4 to 20 kDa range. Spectra were calibrated using Peptide and Protein I Calibrant (Bruker Daltonics, Billerica, Massachusetts, USA). Matrix only and trypsin digest reaction mixture without SOD1 spectra were acquired as negative controls. Spectra were analyzed in flexAnalysis and BioTools 3.2 (Bruker Daltonics, Billerica, Massachusetts, USA). Peptide mass fingerprinting was performed using MASCOT (Matrix Science, Boston, Massachusetts, USA) using trypsin as the enzyme with up to 5 missed cleavages, 100 ppm mass tolerance, and cysteine carbamidomethylation as a variable modification.

### Survival analysis

Two fALS lines were used to assess survival in congenic (later onset at approximately 155 days) and hybrid (earlier onset at approximately 125 days) backgrounds. The first line was derived by crossing (over >10 generations) congenic fALS “B6 G93A mice” (B6.Cg-Tg(SOD1*G93A)1Gur/J, Stock No. 004435, The Jackson Laboratory, Bar Harbor, Maine, USA) with “YFP-16 mice” [50] (B6.Cg-Tg(Thy1-YFP)16Jrs/J, Stock No. 003709, The Jackson Laboratory, Bar Harbor, Maine, USA). This line maintains the C57BL/6J genetic background and expresses YFP in motor and sensory neurons to enable cellular-level molecular profiling [51] in future studies. The second line is the standard “Hybrid B6SJL G93A mice” (B6SJL-Tg(SOD1*G93A)1Gur/J Stock No. 002726, The Jackson Laboratory, Bar Harbor, Maine, USA). Methods for survival studies have been described [79] and followed the conventions for optimal trial design with fALS mice. Mice were tested for difference in: (1) disease onset (at least a 20% decrease in grip strength and open-field motor performance, which we expect to correlate with change in weight); (2) disease end-stage (disease score, e.g., loss of righting reflex); and (3) weight. The hybrid line was additionally tested for differences in forelimb and hindlimb grip strength.

## Supporting information

S1 Fig *S*-XL6 cross-linking of SOD1 variants regulates the structure making them more like the stable, dimeric *wild-type* form. (DOCX)

S2 Fig *S*-XL6 has stabilizing structural effects on SOD1 fALS variants. (DOCX)

S3 Fig Viability and inclusion formation for EGFP-G93A and EGFP-WT-SOD1. (DOCX)

S4 Fig 1H NMR of *S*-XL6. (DOCX)

S5 Fig ^13^C NMR of *S*-XL6. (DOCX)

S6 Fig High-resolution mass spectrum of *S*-XL6 showing observed [M+H]^+^ mass 137.00914 Da. (DOCX)

S7 Fig Bruce’s up-down acute toxicity (LD_50_) analysis of *S*-XL6 in C57BL/6 mice. (DOCX)

S8 Fig Body weight, disease score, and all paws’ grip score analysis using Hybrid B6SJL G93A mice. (DOCX)

S9 Fig Unannotated version of the size exclusion chromatography (as shown before as Fig 3) demonstrates that the monomeric population of fALS variants is decreased by *S*-XL6 treatment and that this is associated with reduced aggregation. (DOCX)

S1 Table Chemical-based enzymatic activity of SOD1 variants. (DOCX)

S2 Table X-ray Crystallographic data collection and refinement statistics. (DOCX)

S3 Table Pharmacodynamic analysis of *S*-XL6 cross-linked SOD1^G93A^ dimer. (DOCX)

S4 Table *S*-XL6 does not alter the metal binding affinity for SOD1 variants. (DOCX)

S1 Data All experimental raw data in an Excel spreadsheet. (XLSX)

S1 Raw Images All uncut raw gel and blot figures. (PDF)

## Abbreviations

ALS: amyotrophic lateral sclerosis
DSF: differential scanning fluorimetry
fALS: familial amyotrophic lateral sclerosis
FBS: fetal bovine serum
FT-ICR-MS: Fourier-transform ion cyclotron resonance mass spectrometry
H/D-X MS: hydrogen deuterium exchange mass spectrometry
IV: intravenous
LC-MS: liquid chromatography-mass spectrometry
LC-MS/MS: liquid chromatography-tandem mass spectrometry
MALDI: matrix-assisted laser desorption ionization
MS: mass spectrometry
NBT: nitroblue tetrazolium
NCI: National Cancer Institute
PD: pharmacodynamic
PTM: posttranslational modification
RBC: red blood cell
sALS: sporadic amyotrophic lateral sclerosis
SARS-CoV-2: Severe Acute Respiratory Syndrome Coronavirus 2
SAXS: small-angle X-ray scattering
SC: subcutaneous
SEC: size exclusion chromatography
SOD1: superoxide dismutase 1

## References

[1] Abalkhail H, Mitchell J, Habgood J, Orrell R, de Belleroche J. A new familial amyotrophic lateral sclerosis locus on chromosome 16q12.1-16q12.2. Am J Hum Genet. 2003;73:383–389. doi: 10.1086/377156 12830400 PMC1180375

[2] Chiò A, Logroscino G, Hardiman O, Swingler R, Mitchell D, Beghi E, et al. Prognostic factors in ALS: A critical review. Amyotroph Lateral Scler. 2009;10:310–323. doi: 10.3109/17482960802566824 19922118 PMC3515205

[3] Henden L, Twine NA, Szul P, McCann EP, Nicholson GA, Rowe DB, et al. Identity by descent analysis identifies founder events and links SOD1 familial and sporadic ALS cases. NPJ Genom Med. 2020;5:32. doi: 10.1038/s41525-020-00139-8 32789025 PMC7414871

[4] Renton AE, Chiò A, Traynor BJ. State of play in amyotrophic lateral sclerosis genetics. Nat Neurosci. 2014;17:17–23. doi: 10.1038/nn.3584 24369373 PMC4544832

[5] Deng HX, Hentati A, Tainer JA, Iqbal Z, Cayabyab A, Hung WY, et al. Amyotrophic lateral sclerosis and structural defects in Cu,Zn superoxide dismutase. Science. 1993;261:1047. doi: 10.1126/science.8351519 8351519

[6] Guissart C, Mouzat K, Kantar J, Louveau B, Vilquin P, Polge A, et al. Premature termination codons in SOD1 causing Amyotrophic Lateral Sclerosis are predicted to escape the nonsense-mediated mRNA decay. Sci Rep. 2020;10:20738. doi: 10.1038/s41598-020-77716-5 33244158 PMC7691510

[7] Valentine JS, Hart PJ. Misfolded CuZnSOD and amyotrophic lateral sclerosis. Proc Natl Acad Sci U S A. 2003;100:3617–3622. doi: 10.1073/pnas.0730423100 12655070 PMC152971

[8] Rosen DR, Siddique T, Patterson D, Figlewicz DA, Sapp P, Hentati A, et al. Mutations in Cu/Zn superoxide dismutase gene are associated with familial amyotrophic lateral sclerosis. Nature. 1993;362:59–62. doi: 10.1038/362059a0 8446170

[9] Wang Q, Johnson JL, Agar NY, Agar JN. Protein aggregation and protein instability govern familial amyotrophic lateral sclerosis patient survival. PLoS Biol. 2008;6:e170. doi: 10.1371/journal.pbio.0060170 18666828 PMC2486295

[10] Bosco DA, Morfini G, Karabacak NM, Song Y, Gros-Louis F, Pasinelli P, et al. Wild-type and mutant SOD1 share an aberrant conformation and a common pathogenic pathway in ALS. Nat Neurosci. 2010;13:1396–1403. doi: 10.1038/nn.2660 20953194 PMC2967729

[11] Maier M, Welt T, Wirth F, Montrasio F, Preisig D, McAfoose J, et al. A human-derived antibody targets misfolded SOD1 and ameliorates motor symptoms in mouse models of amyotrophic lateral sclerosis. Sci Transl Med. 2018;10:eaah3924. doi: 10.1126/scitranslmed.aah3924 30518612

[12] Furukawa Y, Tokuda E. Does wild-type Cu/Zn-superoxide dismutase have pathogenic roles in amyotrophic lateral sclerosis? Transl Neurodegener. 2020;9:33. doi: 10.1186/s40035-020-00209-y 32811540 PMC7437001

[13] Gurney ME, Pu H, Chiu AY, Dal Canto MC, Polchow CY, Alexander DD, et al. Motor neuron degeneration in mice that express a human Cu,Zn superoxide dismutase mutation. Science. 1994;264:1772–1775. doi: 10.1126/science.8209258 8209258

[14] Wright GS, Antonyuk SV, Kershaw NM, Strange RW, Samar Hasnain S. Ligand binding and aggregation of pathogenic SOD1. Nat Commun. 2013;4:1758. doi: 10.1038/ncomms2750 23612299 PMC3644087

[15] Nowak RJ, Cuny GD, Choi S, Lansbury PT, Ray SS. Improving binding specificity of pharmacological chaperones that target mutant superoxide dismutase-1 linked to familial amyotrophic lateral sclerosis using computational methods. J Med Chem. 2010;53:2709–2718. doi: 10.1021/jm901062p 20232802 PMC2881568

[16] Maurer MS, Schwartz JH, Gundapaneni B, Elliott PM, Merlini G, Waddington-Cruz M, et al. Tafamidis Treatment for Patients with Transthyretin Amyloid Cardiomyopathy. N Engl J Med. 2018;379:1007–1016. doi: 10.1056/NEJMoa1805689 30145929

[17] Ray SS, Nowak RJ, Brown RH Jr, Lansbury PT Jr. Small-molecule-mediated stabilization of familial amyotrophic lateral sclerosis-linked superoxide dismutase mutants against unfolding and aggregation. Proc Natl Acad Sci U S A. 2005;102:3639–3644. doi: 10.1073/pnas.0408277102 15738401 PMC553303

[18] Koppaka V, Thompson DC, Chen Y, Ellermann M, Nicolaou KC, Juvonen RO, et al. Aldehyde dehydrogenase inhibitors: a comprehensive review of the pharmacology, mechanism of action, substrate specificity, and clinical application. Pharmacol Rev. 2012;64:520–539. doi: 10.1124/pr.111.005538 22544865 PMC3400832

[19] Olbe L, Carlsson E, Lindberg P. A proton-pump inhibitor expedition: the case histories of omeprazole and esomeprazole. Nat Rev Drug Discov. 2003;2:132–139. doi: 10.1038/nrd1010 12563304

[20] Amporndanai K, Meng X, Shang W, Jin Z, Rogers M, Zhao Y, et al. Inhibition mechanism of SARS-CoV-2 main protease by ebselen and its derivatives. Nat Commun. 2021;12:3061. doi: 10.1038/s41467-021-23313-7 34031399 PMC8144557

[21] Li D, Ambrogio L, Shimamura T, Kubo S, Takahashi M, Chirieac LR, et al. BIBW2992, an irreversible EGFR/HER2 inhibitor highly effective in preclinical lung cancer models. Oncogene. 2008;27:4702–4711. doi: 10.1038/onc.2008.109 18408761 PMC2748240

[22] Auclair JR, Boggio KJ, Petsko GA, Ringe D, Agar JN. Strategies for stabilizing superoxide dismutase (SOD1), the protein destabilized in the most common form of familial amyotrophic lateral sclerosis. Proc Natl Acad Sci U S A. 2010;107:21394–21399. doi: 10.1073/pnas.1015463107 21098299 PMC3003092

[23] Cooney DA, Milman HA, Cable RG, Dion RL, Bono VH Jr. Maleimide—biochemical, pharmacologic and toxicologic studies. Interaction with L-asparagine metabolism. Biochem Pharmacol. 1978;27:151–166. doi: 10.1016/0006-2952(78)90295-2 23785

[24] Capper MJ, Wright GSA, Barbieri L, Luchinat E, Mercatelli E, McAlary L, et al. The cysteine-reactive small molecule ebselen facilitates effective SOD1 maturation. Nat Commun. 2018;9:1693. doi: 10.1038/s41467-018-04114-x 29703933 PMC5923229

[25] Banci L, Bertini I, Blaževitš O, Calderone V, Cantini F, Mao J, et al. Interaction of cisplatin with human superoxide dismutase. J Am Chem Soc. 2012;134:7009–7014. doi: 10.1021/ja211591n 22471402

[26] Amporndanai K, Rogers M, Watanabe S, Yamanaka K, O’Neill PM, Hasnain SS. Novel Selenium-based compounds with therapeutic potential for SOD1-linked amyotrophic lateral sclerosis. EBioMedicine. 2020;59:102980. doi: 10.1016/j.ebiom.2020.102980 32862101 PMC7456458

[27] Chantadul V, Wright GSA, Amporndanai K, Shahid M, Antonyuk SV, Washbourn G, et al. Ebselen as template for stabilization of A4V mutant dimer for motor neuron disease therapy. Commun Biol. 2020;3:97. doi: 10.1038/s42003-020-0826-3 32139772 PMC7058017

[28] Donnelly DP, Dowgiallo MG, Salisbury JP, Aluri KC, Iyengar S, Chaudhari M, et al. Cyclic Thiosulfinates and Cyclic Disulfides Selectively Cross-Link Thiols While Avoiding Modification of Lone Thiols. J Am Chem Soc. 2018;140:7377–7380. doi: 10.1021/jacs.8b01136 29851341

[29] Cropley TC, Liu FC, Pedrete T, Hossain MA, Agar JN, Bleiholder C. Structure Relaxation Approximation (SRA) for Elucidation of Protein Structures from Ion Mobility Measurements (II). Protein Complexes. J Phys Chem B. 2023;127:5553–5565. doi: 10.1021/acs.jpcb.3c01024 37311097

[30] Stathopulos PB, Rumfeldt JA, Scholz GA, Irani RA, Frey HE, Hallewell RA, et al. Cu/Zn superoxide dismutase mutants associated with amyotrophic lateral sclerosis show enhanced formation of aggregates in vitro. Proc Natl Acad Sci U S A. 2003;100:7021–7026. doi: 10.1073/pnas.1237797100 12773627 PMC165823

[31] Niesen FH, Berglund H, Vedadi M. The use of differential scanning fluorimetry to detect ligand interactions that promote protein stability. Nat Protoc. 2007;2:2212–2221. doi: 10.1038/nprot.2007.321 17853878

[32] Liu Y-T, Yen Y-J, Ricardo F, Chang Y, Wu P-H, Huang S-J, et al. Biophysical characterization and modulation of Transthyretin Ala97Ser. Ann Clin Transl Neurol. 2019;6:1961–1970. doi: 10.1002/acn3.50887 31502419 PMC6801203

[33] McAlary L, Shephard VK, Wright GSA, Yerbury JJ. A copper chaperone-mimetic polytherapy for SOD1-associated amyotrophic lateral sclerosis. J Biol Chem. 2022;298:101612. doi: 10.1016/j.jbc.2022.101612 35065969 PMC8885447

[34] Wilcox KC, Zhou L, Jordon JK, Huang Y, Yu Y, Redler RL, et al. Modifications of superoxide dismutase (SOD1) in human erythrocytes: a possible role in amyotrophic lateral sclerosis. J Biol Chem. 2009;284:13940–13947. doi: 10.1074/jbc.M809687200 19299510 PMC2679493

[35] Gabrielson JP, Brader ML, Pekar AH, Mathis KB, Winter G, Carpenter JF, et al. Quantitation of aggregate levels in a recombinant humanized monoclonal antibody formulation by size-exclusion chromatography, asymmetrical flow field flow fractionation, and sedimentation velocity. J Pharm Sci. 2007;96:268–279. doi: 10.1002/jps.20760 17080424

[36] Fekete S, Beck A, Veuthey JL, Guillarme D. Theory and practice of size exclusion chromatography for the analysis of protein aggregates. J Pharm Biomed Anal. 2014;101:161–173. doi: 10.1016/j.jpba.2014.04.011 24816223

[37] Redler RL, Fee L, Fay JM, Caplow M, Dokholyan NV. Non-native soluble oligomers of Cu/Zn superoxide dismutase (SOD1) contain a conformational epitope linked to cytotoxicity in amyotrophic lateral sclerosis (ALS). Biochemistry. 2014;53:2423–2432. doi: 10.1021/bi500158w 24660965 PMC4004233

[38] Rasouli S, Abdolvahabi A, Croom CM, Plewman DL, Shi Y, Shaw BF. Glycerolipid Headgroups Control Rate and Mechanism of Superoxide Dismutase-1 Aggregation and Accelerate Fibrillization of Slowly Aggregating Amyotrophic Lateral Sclerosis Mutants. ACS Chem Nerosci. 2018;9:1743–1756. doi: 10.1021/acschemneuro.8b00086 29649360

[39] Rasouli S, Abdolvahabi A, Croom CM, Plewman DL, Shi Y, Ayers JI, et al. Lysine acylation in superoxide dismutase-1 electrostatically inhibits formation of fibrils with prion-like seeding. J Biol Chem. 2017;292:19366–19380. doi: 10.1074/jbc.M117.805283 28974578 PMC5702675

[40] Wales TE, Engen JR. Hydrogen exchange mass spectrometry for the analysis of protein dynamics. Mass Spectrom Rev. 2006;25:158–170. doi: 10.1002/mas.20064 16208684

[41] Elam JS, Taylor AB, Strange R, Antonyuk S, Doucette PA, Rodriguez JA, et al. Amyloid-like filaments and water-filled nanotubes formed by SOD1 mutant proteins linked to familial ALS. Nat Struct Biol. 2003;10:461–467. doi: 10.1038/nsb935 12754496

[42] McAlary L, Aquilina JA, Yerbury JJ. Susceptibility of Mutant SOD1 to Form a Destabilized Monomer Predicts Cellular Aggregation and Toxicity but Not In vitro Aggregation Propensity. Front Neurosci. 2016;10:499. doi: 10.3389/fnins.2016.00499 27867347 PMC5095133

[43] Ip P, Mulligan VK, Chakrabartty A. ALS-causing SOD1 mutations promote production of copper-deficient misfolded species. J Mol Biol. 2011;409:839–852. doi: 10.1016/j.jmb.2011.04.027 21549128

[44] Hayward LJ, Rodriguez JA, Kim JW, Tiwari A, Goto JJ, Cabelli DE, et al. Decreased metallation and activity in subsets of mutant superoxide dismutases associated with familial amyotrophic lateral sclerosis. J Biol Chem. 2002;277:15923–15931. doi: 10.1074/jbc.M112087200 11854284

[45] Aluri KC, Hossain MA, Kanetkar N, Miller BC, Dowgiallo MG, Sivasankar D, et al. Cyclic Thiosulfinates as a Novel Class of Disulfide Cleavable Cross-Linkers for Rapid Hydrogel Synthesis. Bioconjug Chem. 2021;32:584–594. doi: 10.1021/acs.bioconjchem.1c00049 33606505

[46] Nakashima F, Shibata T, Kamiya K, Yoshitake J, Kikuchi R, Matsushita T, et al. Structural and functional insights into S-thiolation of human serum albumins. Sci Rep. 2018;8:932. doi: 10.1038/s41598-018-19610-9 29343798 PMC5772555

[47] Pratt AJ, Shin DS, Merz GE, Rambo RP, Lancaster WA, Dyer KN, et al. Aggregation propensities of superoxide dismutase G93 hotspot mutants mirror ALS clinical phenotypes. Proc Natl Acad Sci U S A. 2014;111:E4568. doi: 10.1073/pnas.1308531111 25316790 PMC4217430

[48] McCord JM, Fridovich I. Superoxide dismutase. An enzymic function for erythrocuprein (hemocuprein). J Biol Chem. 1969;244:6049–6055. 5389100

[49] Kabuta T, Suzuki Y, Wada K. Degradation of Amyotrophic Lateral Sclerosis-linked Mutant Cu,Zn-Superoxide Dismutase Proteins by Macroautophagy and the Proteasome. J Biol Chem. 2006;281:30524–30533. doi: 10.1074/jbc.M603337200 16920710

[50] Feng G, Mellor RH, Bernstein M, Keller-Peck C, Nguyen QT, Wallace M, et al. Imaging neuronal subsets in transgenic mice expressing multiple spectral variants of GFP. Neuron. 2000;28:41–51. doi: 10.1016/s0896-6273(00)00084-2 11086982

[51] Schmitt ND, Rawlins CM, Randall EC, Wang X, Koller A, Auclair JR, et al. Genetically Encoded Fluorescent Proteins Enable High-Throughput Assignment of Cell Cohorts Directly from MALDI-MS Images. Anal Chem. 2019;91:3810–3817. doi: 10.1021/acs.analchem.8b03454 30839199 PMC6827431

[52] Shaw BF, Lelie HL, Durazo A, Nersissian AM, Xu G, Chan PK, et al. Detergent-insoluble aggregates associated with amyotrophic lateral sclerosis in transgenic mice contain primarily full-length, unmodified superoxide dismutase-1. J Biol Chem. 2008;283:8340–8350. doi: 10.1074/jbc.M707751200 18192269 PMC2276386

[53] Williams JR, Trias E, Beilby PR, Lopez NI, Labut EM, Bradford CS, et al. Copper delivery to the CNS by CuATSM effectively treats motor neuron disease in SOD(G93A) mice co-expressing the Copper-Chaperone-for-SOD. Neurobiol Dis. 2016;89:1–9. doi: 10.1016/j.nbd.2016.01.020 26826269 PMC4785045

[54] Donnelly DP, Rawlins CM, DeHart CJ, Fornelli L, Schachner LF, Lin Z, et al. Best practices and benchmarks for intact protein analysis for top-down mass spectrometry. Nat Methods. 2019;16:587–594. doi: 10.1038/s41592-019-0457-0 31249407 PMC6719561

[55] Nozari A, Akeju O, Mirzakhani H, Eskandar E, Ma Z, Hossain MA, et al. Prolonged therapy with the anticonvulsant carbamazepine leads to increased plasma clearance of fentanyl. J Pharm Pharmacol. 2019;71:982–987. doi: 10.1111/jphp.13079 30793320 PMC7938950

[56] Battye TG, Kontogiannis L, Johnson O, Powell HR, Leslie AG. iMOSFLM: a new graphical interface for diffraction-image processing with MOSFLM. Acta Crystallogr D Biol Crystallogr. 2011;67:271–281. doi: 10.1107/S0907444910048675 21460445 PMC3069742

[57] Vagin A, Teplyakov A. Molecular replacement with MOLREP. Acta Crystallogr D Biol Crystallogr. 2010;66:22–25. doi: 10.1107/S0907444909042589 20057045

[58] Murshudov GN, Skubák P, Lebedev AA, Pannu NS, Steiner RA, Nicholls RA, et al. REFMAC5 for the refinement of macromolecular crystal structures. Acta Crystallogr D Biol Crystallogr. 2011;67:355–367. doi: 10.1107/S0907444911001314 21460454 PMC3069751

[59] Emsley P, Cowtan K. Coot: model-building tools for molecular graphics. Acta Crystallogr D Biol Crystallogr. 2004;60:2126–2132. doi: 10.1107/S0907444904019158 15572765

[60] Chen VB, Arendall WB 3rd, Headd JJ, Keedy DA, Immormino RM, Kapral GJ, et al. MolProbity: all-atom structure validation for macromolecular crystallography. Acta Crystallogr D Biol Crystallogr. 2010;66:12–21. doi: 10.1107/S0907444909042073 20057044 PMC2803126

[61] Malik R, Corrales C, Linsenmeier M, Alalami H, Sepanj N, Bitan G. Examination of SOD1 aggregation modulators and their effect on SOD1 enzymatic activity as a proxy for potential toxicity. FASEB J. 2020;34:11957–11969. doi: 10.1096/fj.202000948R 32701214 PMC7903925

[62] Hossain MA, Tran T, Chen T, Mikus G, Greenblatt DJ. Inhibition of human cytochromes P450 in vitro by ritonavir and cobicistat. J Pharm Pharmacol. 2017;69:1786–1793. doi: 10.1111/jphp.12820 28960344

[63] Hong P, Koza S, Bouvier ES. Size-Exclusion Chromatography for the Analysis of Protein Biotherapeutics and their Aggregates. J Liq Chromatogr Relat Technol. 2012;35:2923–2950. doi: 10.1080/10826076.2012.743724 23378719 PMC3556795

[64] Algeelani S, Alam N, Hossain MA, Mikus G, Greenblatt DJ. In vitro inhibition of human UGT isoforms by ritonavir and cobicistat. Xenobiotica. 2018;48:764–769. doi: 10.1080/00498254.2017.1370655 28891378

[65] Cashman NR, Durham HD, Blusztajn JK, Oda K, Tabira T, Shaw IT, et al. Neuroblastoma x spinal cord (NSC) hybrid cell lines resemble developing motor neurons. Dev Dyn. 1992;194:209–221. doi: 10.1002/aja.1001940306 1467557

[66] Rueden CT, Schindelin J, Hiner MC, DeZonia BE, Walter AE, Arena ET, et al. ImageJ2: ImageJ for the next generation of scientific image data. BMC Bioinformatics. 2017;18:529. doi: 10.1186/s12859-017-1934-z 29187165 PMC5708080

[67] Dao D, Fraser AN, Hung J, Ljosa V, Singh S, Carpenter AE. CellProfiler Analyst: interactive data exploration, analysis and classification of large biological image sets. Bioinformatics. 2016;32:3210–3212. doi: 10.1093/bioinformatics/btw390 27354701 PMC5048071

[68] Stopka SA, Ruiz D, Baquer G, Bodineau C, Hossain MA, Pellens VT, et al. Chemical QuantArray: A Quantitative Tool for Mass Spectrometry Imaging. Anal Chem. 2023;95:11243–11253. doi: 10.1021/acs.analchem.3c00803 37469028 PMC10445330

[69] Miller A, York EM, Stopka SA, Martínez-François JR, Hossain MA, Baquer G, et al. Spatially resolved metabolomics and isotope tracing reveal dynamic metabolic responses of dentate granule neurons with acute stimulation. Nat Metab. 2023;5:1820–1835. doi: 10.1038/s42255-023-00890-z 37798473 PMC10626993

[70] Packer MR, Parker JA, Chung JK, Li Z, Lee YK, Cookis T, et al. Raf promotes dimerization of the Ras G-domain with increased allosteric connections. Proc Natl Acad Sci U S A. 2021;118. doi: 10.1073/pnas.2015648118 33653954 PMC7958358

[71] Bruce RD. An up-and-down procedure for acute toxicity testing. Fundam Appl Toxicol. 1985;5:151–157. doi: 10.1016/0272-0590(85)90059-4 3987991

[72] Manalastas-Cantos K, Konarev PV, Hajizadeh NR, Kikhney AG, Petoukhov MV, Molodenskiy DS, et al. ATSAS 3.0: expanded functionality and new tools for small-angle scattering data analysis. J Appl Cryst. 2021;54:343–355. doi: 10.1107/S1600576720013412 33833657 PMC7941305

[73] Pettersen EF, Goddard TD, Huang CC, Couch GS, Greenblatt DM, Meng EC, et al. UCSF Chimera—a visualization system for exploratory research and analysis. J Comput Chem. 2004;25:1605–1612. doi: 10.1002/jcc.20084 15264254

[74] Masson GR, Burke JE, Ahn NG, Anand GS, Borchers C, Brier S, et al. Recommendations for performing, interpreting and reporting hydrogen deuterium exchange mass spectrometry (HDX-MS) experiments. Nat Methods. 2019;16:595–602. doi: 10.1038/s41592-019-0459-y 31249422 PMC6614034

[75] Engen JR, Botzanowski T, Peterle D, Georgescauld F, Wales TE. Developments in Hydrogen/Deuterium Exchange Mass Spectrometry. Anal Chem. 2021;93:567–582. doi: 10.1021/acs.analchem.0c04281 33112590

[76] Mondia JP, Goh F, Bryngelson PA, MacPhee JM, Ali AS, Weiskopf A, et al. Using X-ray fluorescence to measure inorganics in biopharmaceutical raw materials. Anal Methods. 2015;7:3545–3550.

[77] Wang YA, Wu D, Auclair JR, Salisbury JP, Sarin R, Tang Y, et al. Integrated Bottom-Up and Top-Down Liquid Chromatography-Mass Spectrometry for Characterization of Recombinant Human Growth Hormone Degradation Products. Anal Chem. 2017;89:12771–12777. doi: 10.1021/acs.analchem.7b03026 29096433

[78] York EM, Miller A, Stopka SA, Martínez-François JR, Hossain MA, Baquer G, et al. The dentate gyrus differentially metabolizes glucose and alternative fuels during rest and stimulation. J Neurochem. 2023. doi: 10.1111/jnc.16004 37929637 PMC11070451

[79] Keeler AM, Zieger M, Semple C, Pucci L, Veinbachs A, Brown RH Jr, et al. Intralingual and Intrapleural AAV Gene Therapy Prolongs Survival in a SOD1 ALS Mouse Model. Mol Ther Methods Clin Dev. 2020;17:246–257. doi: 10.1016/j.omtm.2019.12.007 31970202 PMC6962641

