# Supplementary material for: Evaluating protein cross-linking as a therapeutic strategy to stabilize SOD1 variants in a mouse model of familial ALS
Source: PLoS Biol. 2024 Jan 30;22(1):e3002462. doi: 10.1371/journal.pbio.3002462 (PMC10826971; doi:10.1371/journal.pbio.3002462)
Supplement: S1 Fig — (DOCX) [file pbio.3002462.s001.docx]

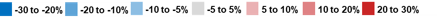


**S1 Fig.** ***S*-XL6 cross-linking of SOD1 variants regulates the structure making them more like the stable, dimeric *wild-type* form**. Differences in deuterium uptake (ΔU, legend shown below) of untreated and crosslinked variants for all timepoints (15s, 50s, 500s, 1 hour, 4 hours) compared to the stable dimeric *wild-type* SOD1 are reported here. For the 4-hr timepoint the prominent ΔU values are as follows: ***N-terminus and nearby:*** *residues 2-7* SOD1^A4V^ untreated 13.8% 🡪 1.2% cross-linked; *residues 2-7* SOD1^G93A^ untreated 3.4% 🡪 -6.2% cross-linked; *residues 2-7* SOD1^G85R^ untreated 5.3% 🡪 -8.4% cross-linked, *residue 8* SOD1^G85R^ untreated 11.9% 🡪 -1.8% cross-linked; *residues 6-7* SOD1^H46R^ untreated 11.5% 🡪 0.6% cross-linked, *residues 9-11* SOD1^H46R^ untreated 17.7% 🡪 6.8% cross-linked. ***Residues 39-43 and nearby****:* SOD1^A4V^ untreated 29% 🡪 17.6% cross-linked. *Residues 22-37,* SOD1^G85R^ untreated 11.9% 🡪 2.9% cross-liked; SOD1^H46R^ untreated 24.6% 🡪 19.7% cross-linked*.* ***Residues 111-116****:* SOD1^G85R^ untreated 9.2% 🡪 -2.0% cross-linked*.* ***C-terminus****:* SOD1^G85R^ untreated 6.2% 🡪 -8.1% cross-linked; SOD1^H46R^ untreated 7.1% 🡪 -5.1% cross-linked. The data underlying this figure can be found in S1_Data.
