# Supplementary material for: Evaluating protein cross-linking as a therapeutic strategy to stabilize SOD1 variants in a mouse model of familial ALS
Source: PLoS Biol. 2024 Jan 30;22(1):e3002462. doi: 10.1371/journal.pbio.3002462 (PMC10826971; doi:10.1371/journal.pbio.3002462)
Supplement: S2 Fig — (DOCX) [file pbio.3002462.s002.docx]

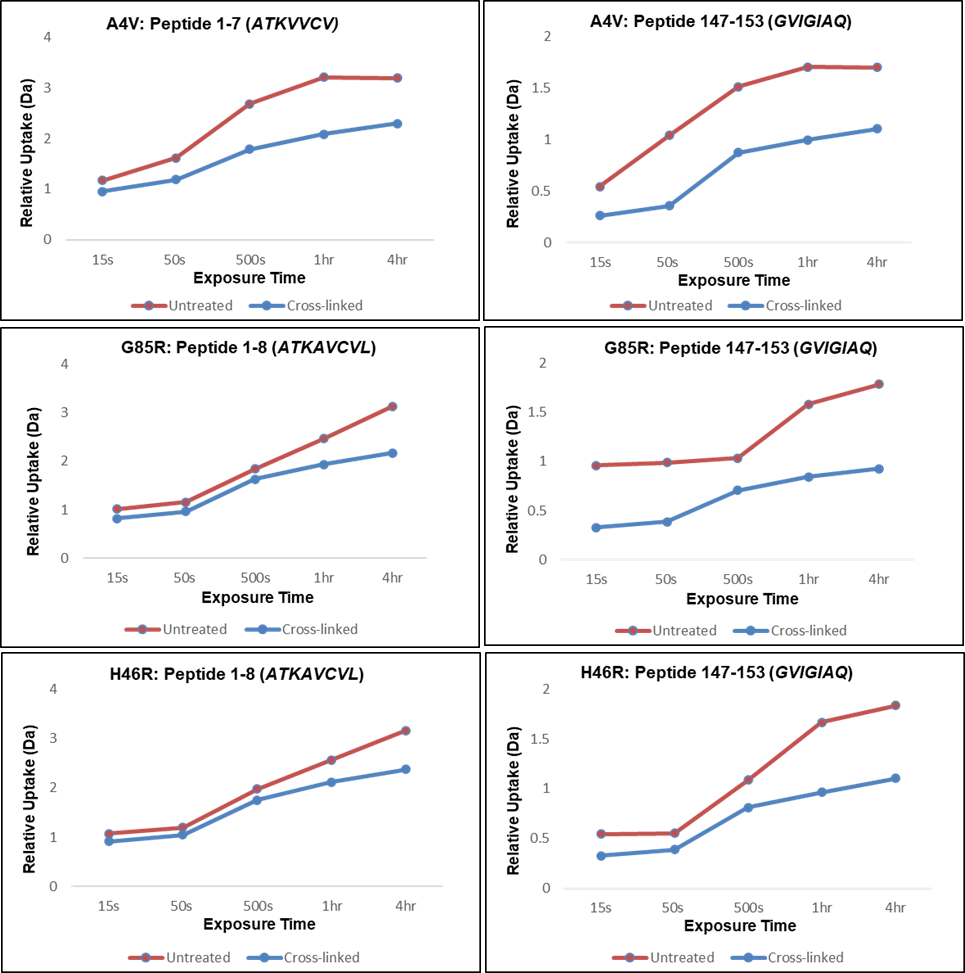


**S2 Fig**. ***S*-XL6 has stabilizing structural effects on SOD1 fALS variants**. Uptake plots for the non-comparable terminal peptides for SOD1^A4V^, SOD1^G85R^, SOD1^H46R^ are shown. Exposure timepoints (15s, 50s, 500s, 1hr, 4hr) are represented on the x-axis plotted against the relative deuterium uptake (Da) on the y-axis. Red lines represent the untreated samples and blue lines represent the *S*-XL6 cross-linked samples. Uptake is generally higher for the control samples, and differences continue to grow with later timepoints (1hr, 4hr). The data underlying this figure can be found in S1_Data.
