# Supplementary material for: Evaluating protein cross-linking as a therapeutic strategy to stabilize SOD1 variants in a mouse model of familial ALS
Source: PLoS Biol. 2024 Jan 30;22(1):e3002462. doi: 10.1371/journal.pbio.3002462 (PMC10826971; doi:10.1371/journal.pbio.3002462)
Supplement: S3 Fig — (DOCX) [file pbio.3002462.s003.docx]

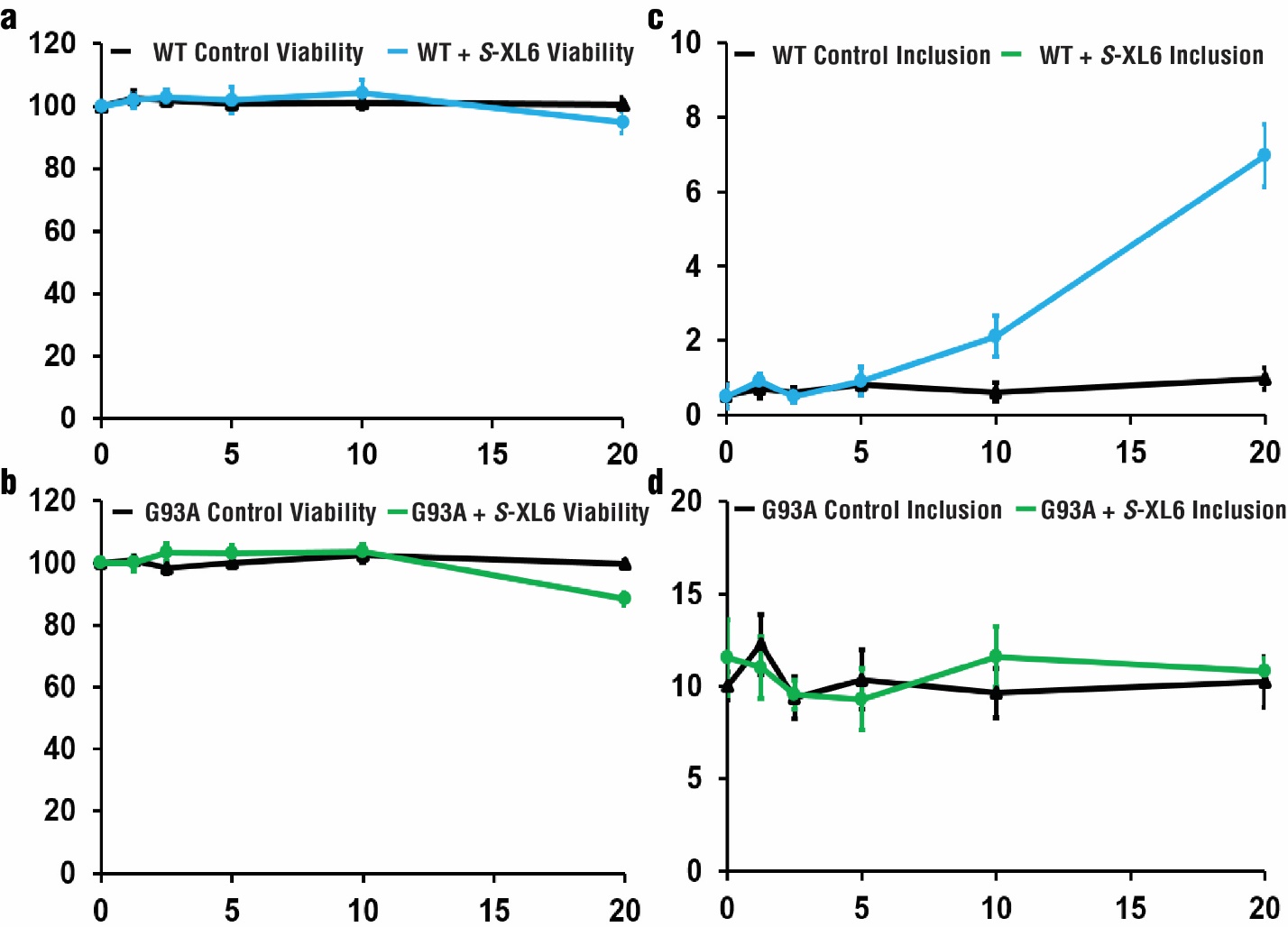


**S3 Fig. Viability and inclusion formation for EGFP-G93A and EGFP-WT-SOD1. Conditions and cell lines were the same used in Fig 7. The data underlying this figure can be found in S1_Data.**
