# Supplementary figures and images for: Evaluating protein cross-linking as a therapeutic strategy to stabilize SOD1 variants in a mouse model of familial ALS
Source: PLoS Biol. 2024 Jan 30;22(1):e3002462. doi: 10.1371/journal.pbio.3002462 (PMC10826971; doi:10.1371/journal.pbio.3002462)

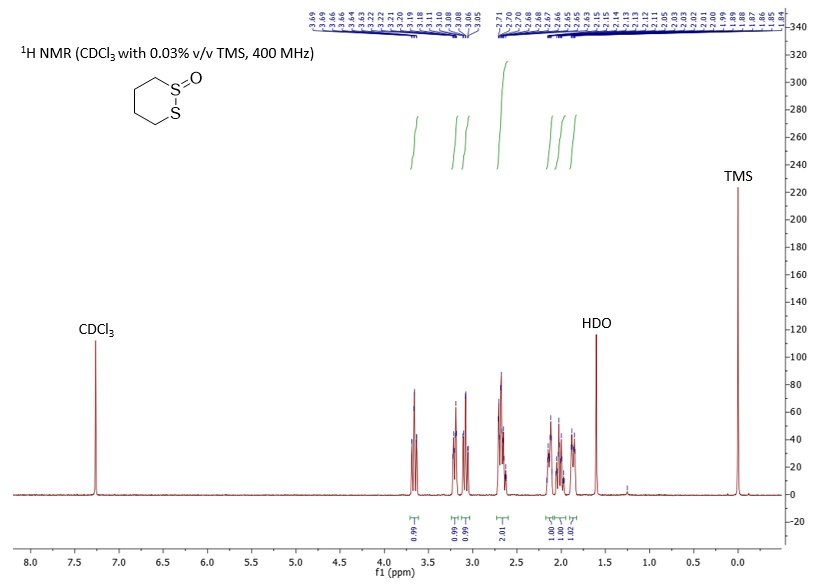


**S4 Fig. 1H NMR of *S*-XL6**.

Supplement: S4 Fig — (DOCX) [file pbio.3002462.s004.docx]

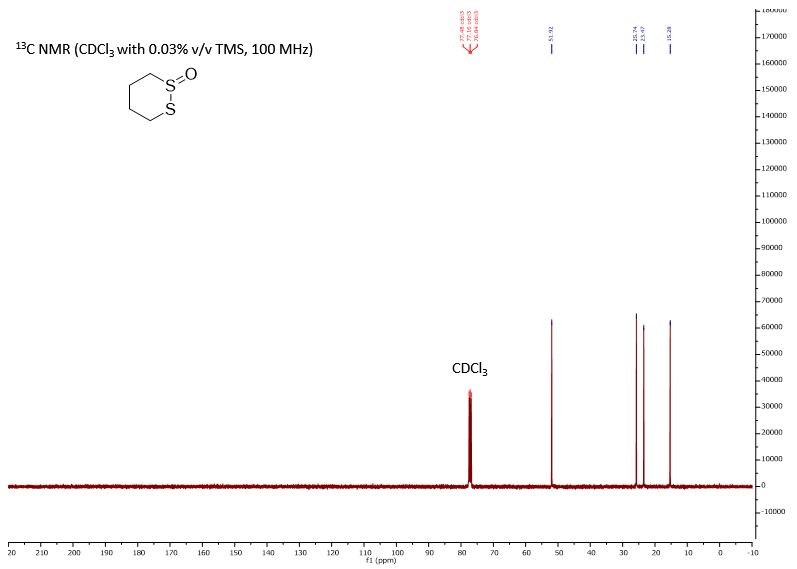


**S5 Fig. 13C NMR of *S*-XL6**.

Supplement: S5 Fig — (DOCX) [file pbio.3002462.s005.docx]

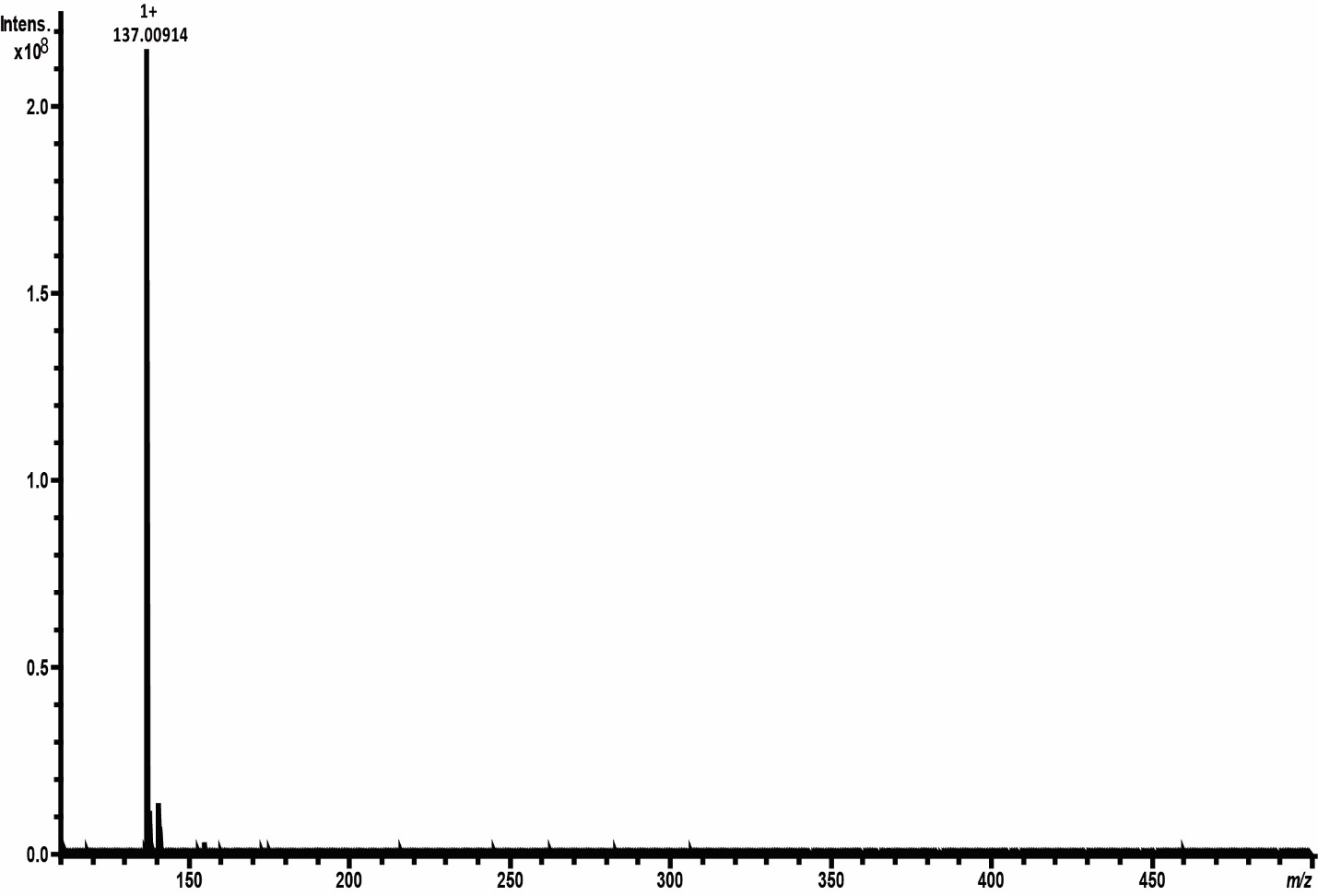


**S6 Fig. High resolution mass spectrum of *S*-XL6 showing observed [M+H]+ mass 137.00914 Da**.

Supplement: S6 Fig — (DOCX) [file pbio.3002462.s006.docx]
