# Supplementary material for: Evaluating protein cross-linking as a therapeutic strategy to stabilize SOD1 variants in a mouse model of familial ALS
Source: PLoS Biol. 2024 Jan 30;22(1):e3002462. doi: 10.1371/journal.pbio.3002462 (PMC10826971; doi:10.1371/journal.pbio.3002462)
Supplement: S7 Fig — (DOCX) [file pbio.3002462.s007.docx]

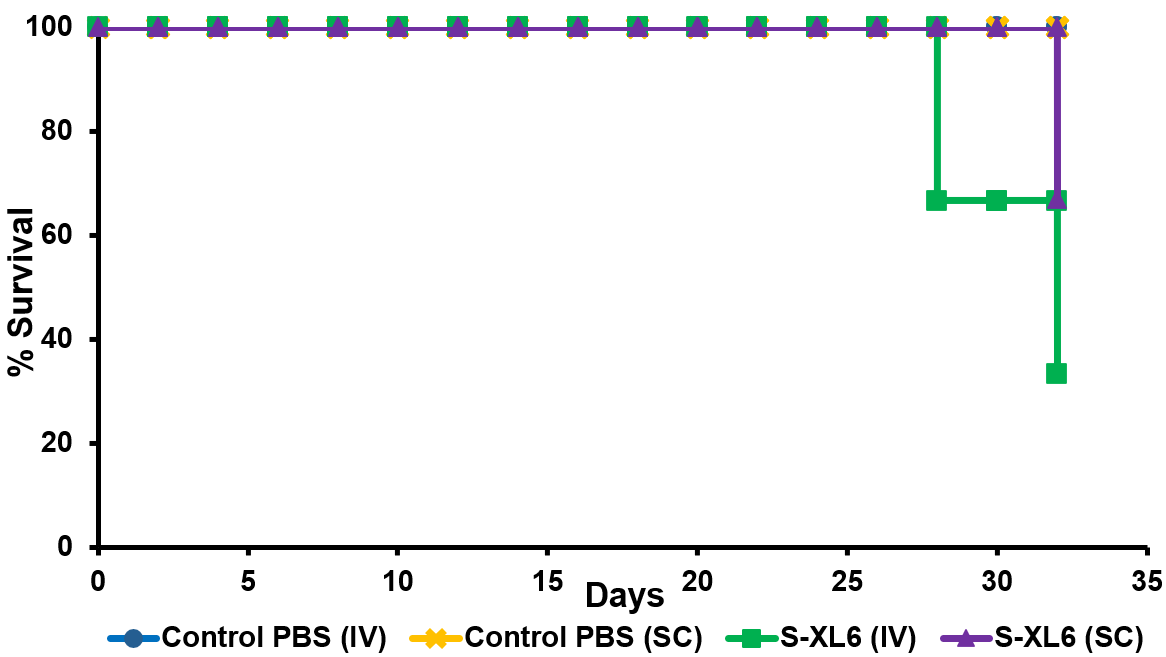


**S7 Fig. Bruce’s up-down acute toxicity (LD_50_) analysis of *S*-XL6 in C57BL/6 mice.** Acute toxicity study was performed using 3 mice for both intravenous (IV) and subcutaneous (SC) group. One mouse died at 55 mg/kg of *S*-XL6 on day 27 and another mouse died at 60 mg/kg of *S*-XL6 on day 32 for IV group. For the SC group, one mouse died at 60 mg/kg of *S*-XL6 on day 32. The data underlying this figure can be found in S1_Data.
