# Supplementary material for: Evaluating protein cross-linking as a therapeutic strategy to stabilize SOD1 variants in a mouse model of familial ALS
Source: PLoS Biol. 2024 Jan 30;22(1):e3002462. doi: 10.1371/journal.pbio.3002462 (PMC10826971; doi:10.1371/journal.pbio.3002462)
Supplement: S8 Fig — (DOCX) [file pbio.3002462.s008.docx]

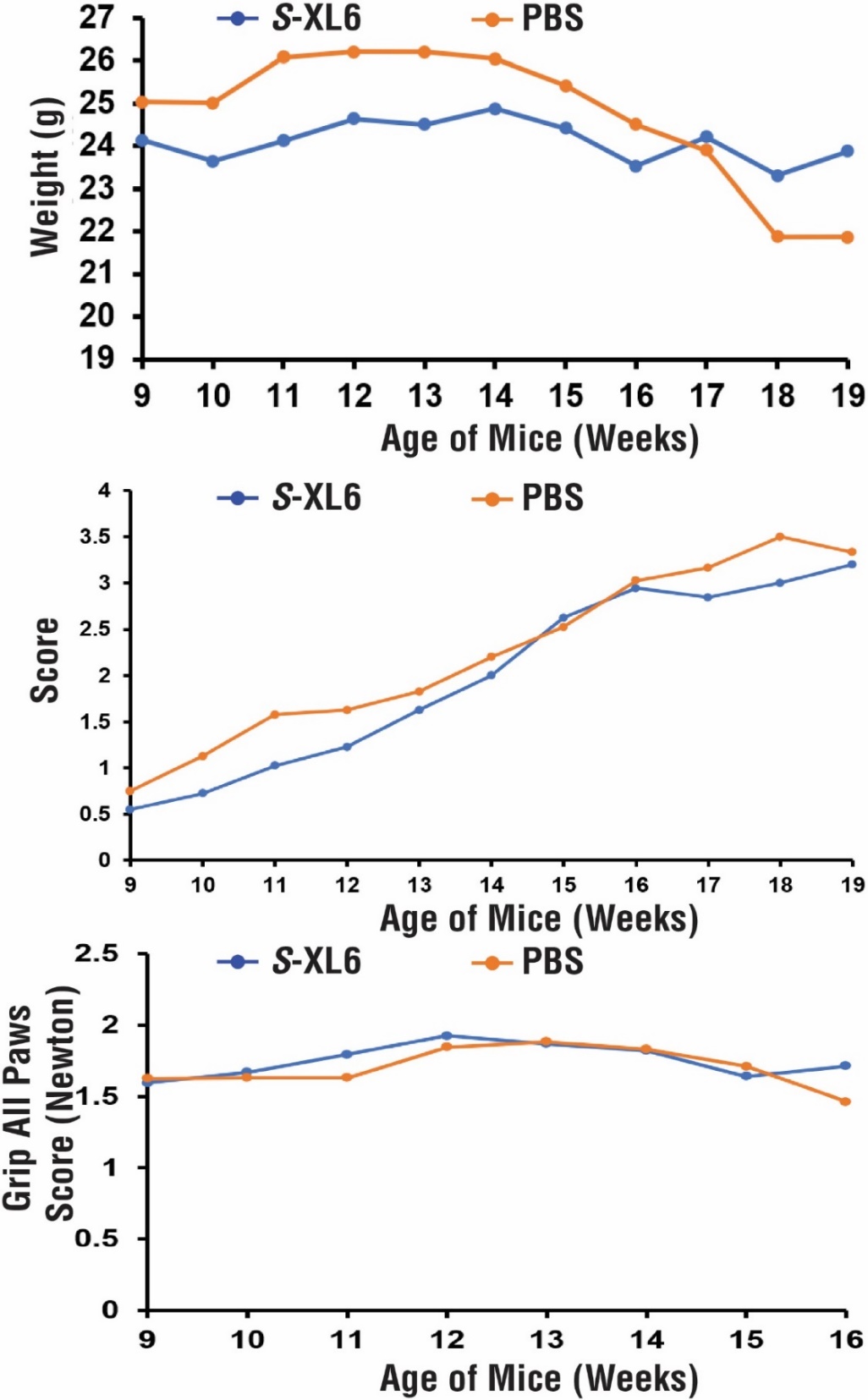


**S8 Fig.** Body weight, disease score, and all paws’ grip score analysis using Hybrid B6SJL G93A mice (B6SJL-Tg(SOD1*G93A)1Gur/J Stock No. 002726, The Jackson Laboratory, Bar Harbor, ME, USA). The data underlying this figure can be found in S1_Data.
