# Supplementary material for: Evaluating protein cross-linking as a therapeutic strategy to stabilize SOD1 variants in a mouse model of familial ALS
Source: PLoS Biol. 2024 Jan 30;22(1):e3002462. doi: 10.1371/journal.pbio.3002462 (PMC10826971; doi:10.1371/journal.pbio.3002462)
Supplement: S9 Fig — (DOCX) [file pbio.3002462.s009.docx]

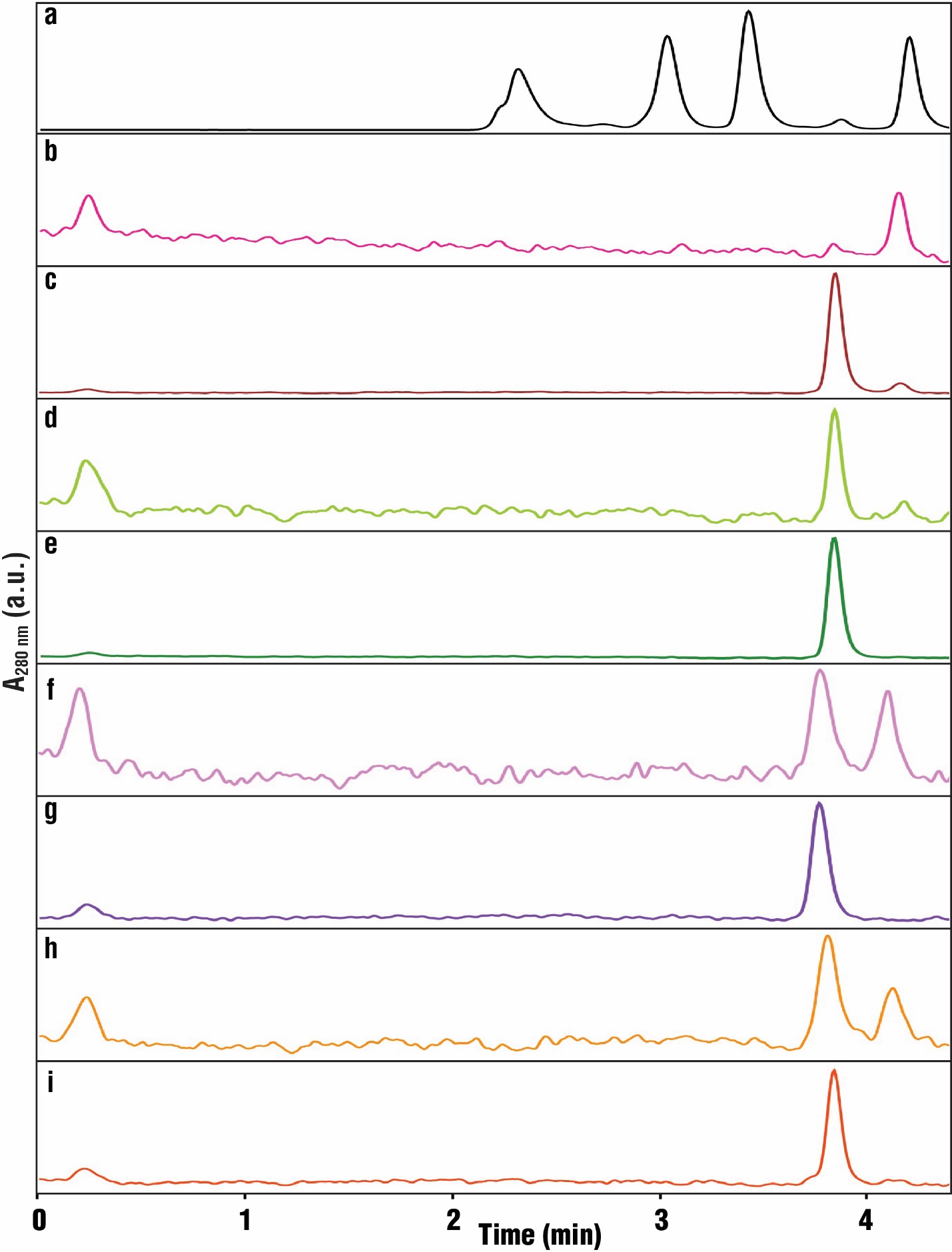


**S9 Fig. Unannotated version of the size exclusion chromatography (as shown before as Fig 3) demonstrates that the monomeric population of fALS variants is decreased by *S*-XL6 treatment and that this is associated with reduced aggregation.**
