# Supplementary material for: Evaluating protein cross-linking as a therapeutic strategy to stabilize SOD1 variants in a mouse model of familial ALS
Source: PLoS Biol. 2024 Jan 30;22(1):e3002462. doi: 10.1371/journal.pbio.3002462 (PMC10826971; doi:10.1371/journal.pbio.3002462)
Supplement: S1 Table — (DOCX) [file pbio.3002462.s010.docx]

| **Sample Type** | **Activity  (U/mL)** | **STD Dev** |
| --- | --- | --- |
| ***Wild-type*** | 135.28 | 6.68 |
| ***Wild-type* + *S*-XL6** | 162.25 | 1.88 |
| **A4V** | 58.86 | 1.37 |
| **A4V + *S*-XL6** | 174.66 | 6.13 |
| **G93A** | 77.96 | 1.25 |
| **G93A + *S*-XL6** | 186.21 | 2.63 |
| **H46R** | -28.09 | 0.30 |
| **H46R + *S*-XL6** | -27.55 | 1.25 |
| **G85R** | 11.89 | 1.75 |
| **G85R + *S*-XL6** | 101.82 | 2.98 |
| **Neg. Control** | 0.00 | 0.31 |

**S1 Table.** **Chemical-based enzymatic activity of SOD1 variants.** Table shows the activity in U/mL unit for SOD1 variants with and without *S*-XL6 treatment. The data underlying this figure can be found in S1_Data.
