# Supplementary material for: Evaluating protein cross-linking as a therapeutic strategy to stabilize SOD1 variants in a mouse model of familial ALS
Source: PLoS Biol. 2024 Jan 30;22(1):e3002462. doi: 10.1371/journal.pbio.3002462 (PMC10826971; doi:10.1371/journal.pbio.3002462)
Supplement: S2 Table — (DOCX) [file pbio.3002462.s011.docx]

|  |  |  |
| --- | --- | --- |
| Data collection  Space group | S-XL6 complex  P21 | As isolated  P21 |
| Resolution (Å) | 37.3 - 1.67 (1.69 - 1.67) | 35.34-1.77 (1.82-1.77) |
| Completeness (%) | 95.2 (93.1) | 97.3(96.9) |
| R merge (%) | 14.6 (47.0) | 14.4(43.9) |
| R pim (%) | 9.1(29.4) | 9.3(29.6) |
| <I>/σI | 83.8(5.2) | 3.9(1.0) |
| Redundancy | 3.4 (3.5) | 3.3(3.3) |
| Overall reflections | 96543 | 82645 |
| Unique reflections | 28274 | 25235 |
| Wilson B-factor (Å^2^) | 14.5 | 10.1 |
| Unit cell (Å, °)  a  b  c  β | 38.81  68.02  50.69  105.85 | 39.26  68.01  52.58  106.28 |
| Solvent (%) | 34 | 34 |
| **Refinement** |  |  |
| No. reflections all/free | 28270 /1400 | 24287/ 933 |
| R-factor/R-free(%) | 18.5 / 23.9 | 21.2/25.7 |
| No. Atoms  Protein/Ions/Ligands/Water  Average B-factor (Å)^2^  Protein/Ligand/Water | 2291/6/25/293  20.07/18.97/38.95/32.8 | 2255/4/19/335  20.47/24.46/39.5/29.85 |
| RMS Deviations |  |  |
| Bonds | 0.0106 | 0.009 |
| Angles | 1.661 | 1.661 |
| PDB ID | 8CCX | 8Q6M |

**S2 Table**. **X-ray Crystallographic data collection and refinement statistics.**
