# Supplementary material for: Evaluating protein cross-linking as a therapeutic strategy to stabilize SOD1 variants in a mouse model of familial ALS
Source: PLoS Biol. 2024 Jan 30;22(1):e3002462. doi: 10.1371/journal.pbio.3002462 (PMC10826971; doi:10.1371/journal.pbio.3002462)
Supplement: S3 Table — (DOCX) [file pbio.3002462.s012.docx]

| **Time (hr.)** | **% Dimer** |
| --- | --- |
| **0.5** | **38** |
| **1** | **63** |
| **2** | **55** |
| **4** | **53** |
| **8** | **51** |
| **12** | **42** |
| **24** | **26** |
| **48** | **20** |
| **72** | **16** |
| **168** | **6** |

S3 Table. Pharmacodynamic analysis of *S*-XL6 cross-linked SOD1^G93A^ dimer. Blood from SOD1^G93A^ mice was collected at different time points post *S*-XL6 dosing at 10 mg/kg via tail vein injection. Table shows the percentage of SOD1^G93A^ cross-linked dimer at different timepoints.
