# Supplementary material for: Evaluating protein cross-linking as a therapeutic strategy to stabilize SOD1 variants in a mouse model of familial ALS
Source: PLoS Biol. 2024 Jan 30;22(1):e3002462. doi: 10.1371/journal.pbio.3002462 (PMC10826971; doi:10.1371/journal.pbio.3002462)
Supplement: S4 Table — (DOCX) [file pbio.3002462.s013.docx]

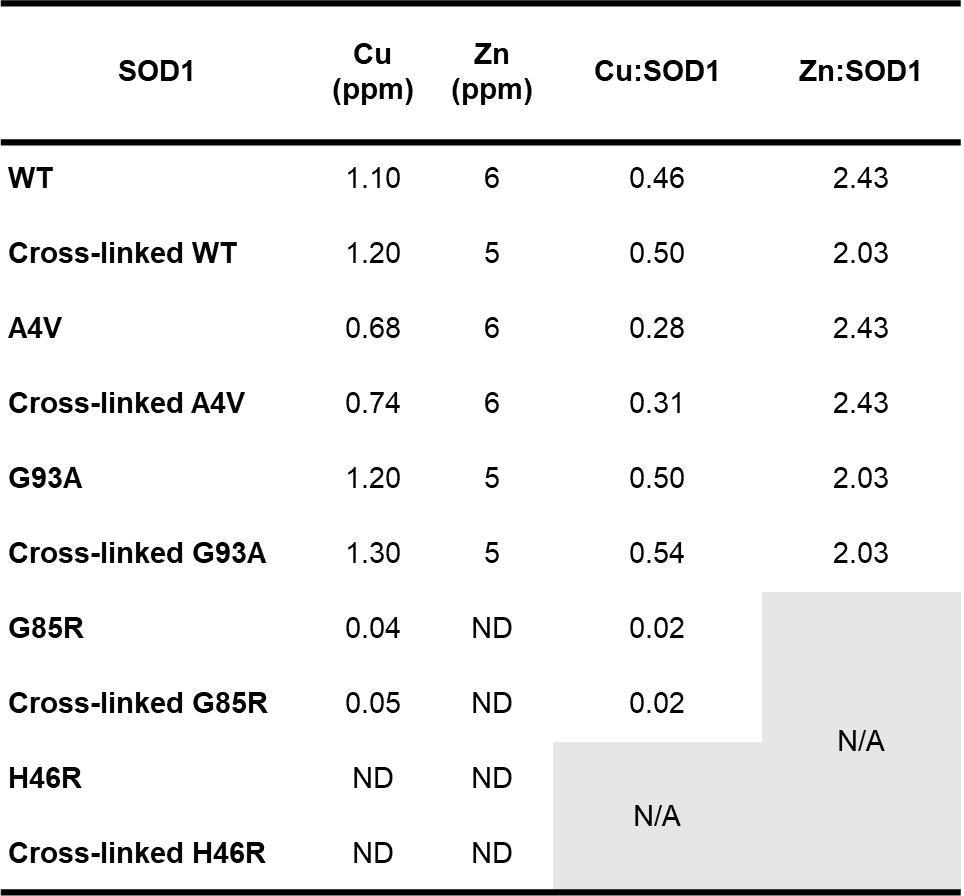


**S4 Table.** ***S*-XL6 does not alter the metal binding affinity for SOD1 variants.** Utilizing ICP-MS, we confirmed the metal content for each SOD1 sample: *wild-type*-like variants (SOD1^A4V^ and SOD1^G93A^) were partially metallated and metal deficient variants (SOD1^G85R^ and SOD1^H46R^) contained little to no metal**.** Metal content is calculated per monomer. The detection limit for copper is 0.02 ppm and Zinc 2 ppm. ND: not detected.
