# Supplementary material for: Evaluating protein cross-linking as a therapeutic strategy to stabilize SOD1 variants in a mouse model of familial ALS
Source: PLoS Biol. 2024 Jan 30;22(1):e3002462. doi: 10.1371/journal.pbio.3002462 (PMC10826971; doi:10.1371/journal.pbio.3002462)
Supplement: S1 Raw Images — (PDF) [file pbio.3002462.s015.pdf]

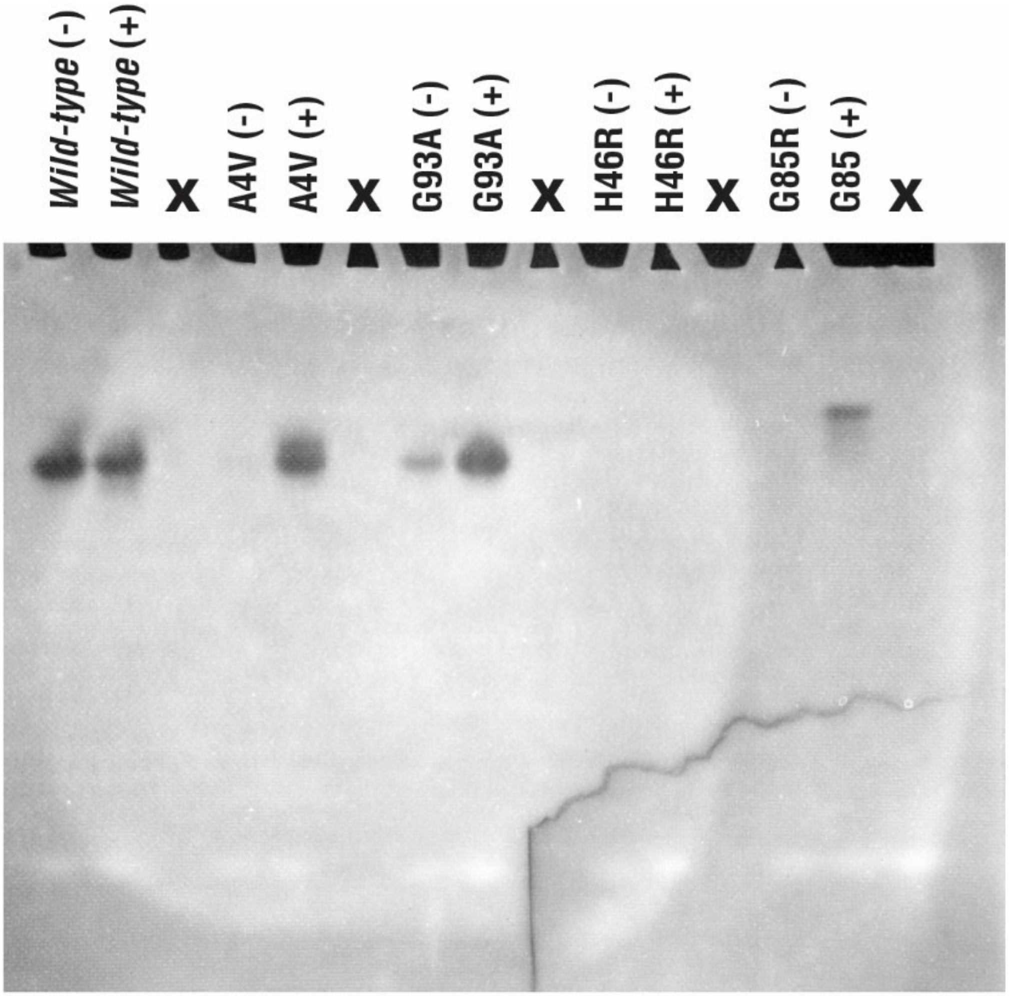

Fig. 5B

***We have not used any molecular weight marker in the activity gel (Fig. 5B) since we tested SOD1 activity only in this experiment, which can be seen as bands for increased activity.*

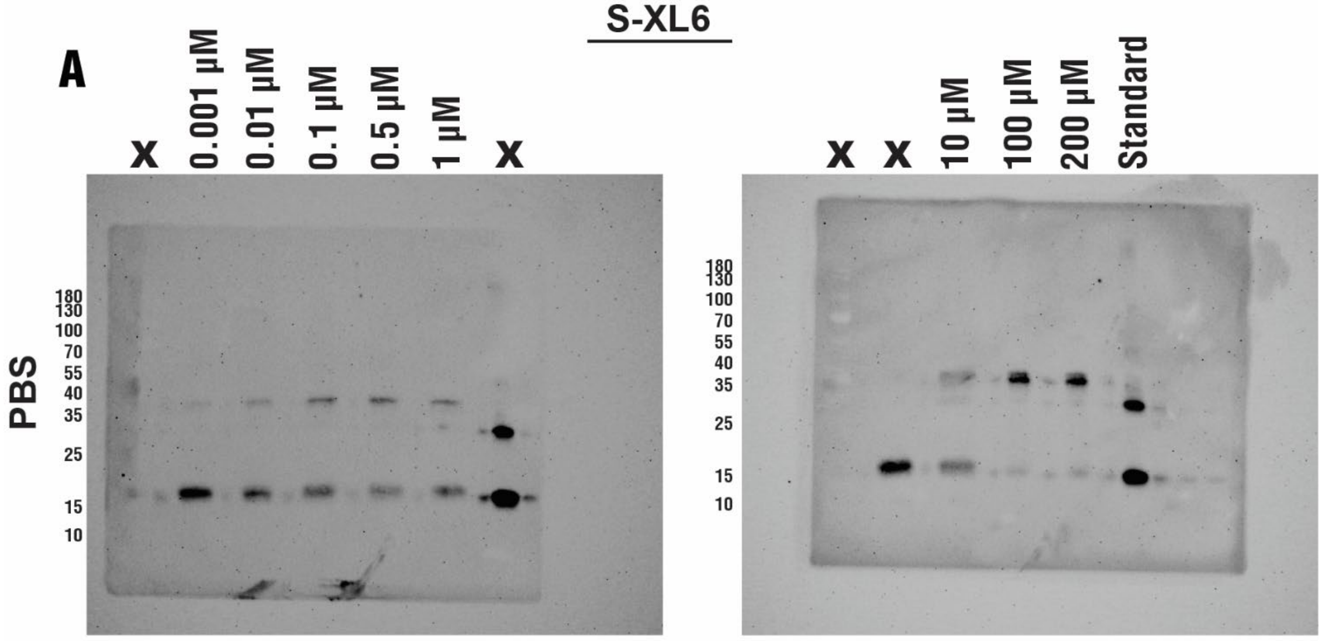
S-XL6
A
PBS
x
0.001 μM
0.01 μM
0.1 μM
0.5 μM
1 μM
x
180
130
100
70
55
40
35
25
15
10
x
x
10 μM
100 μM
200 μM
Standard

Fig. 6A

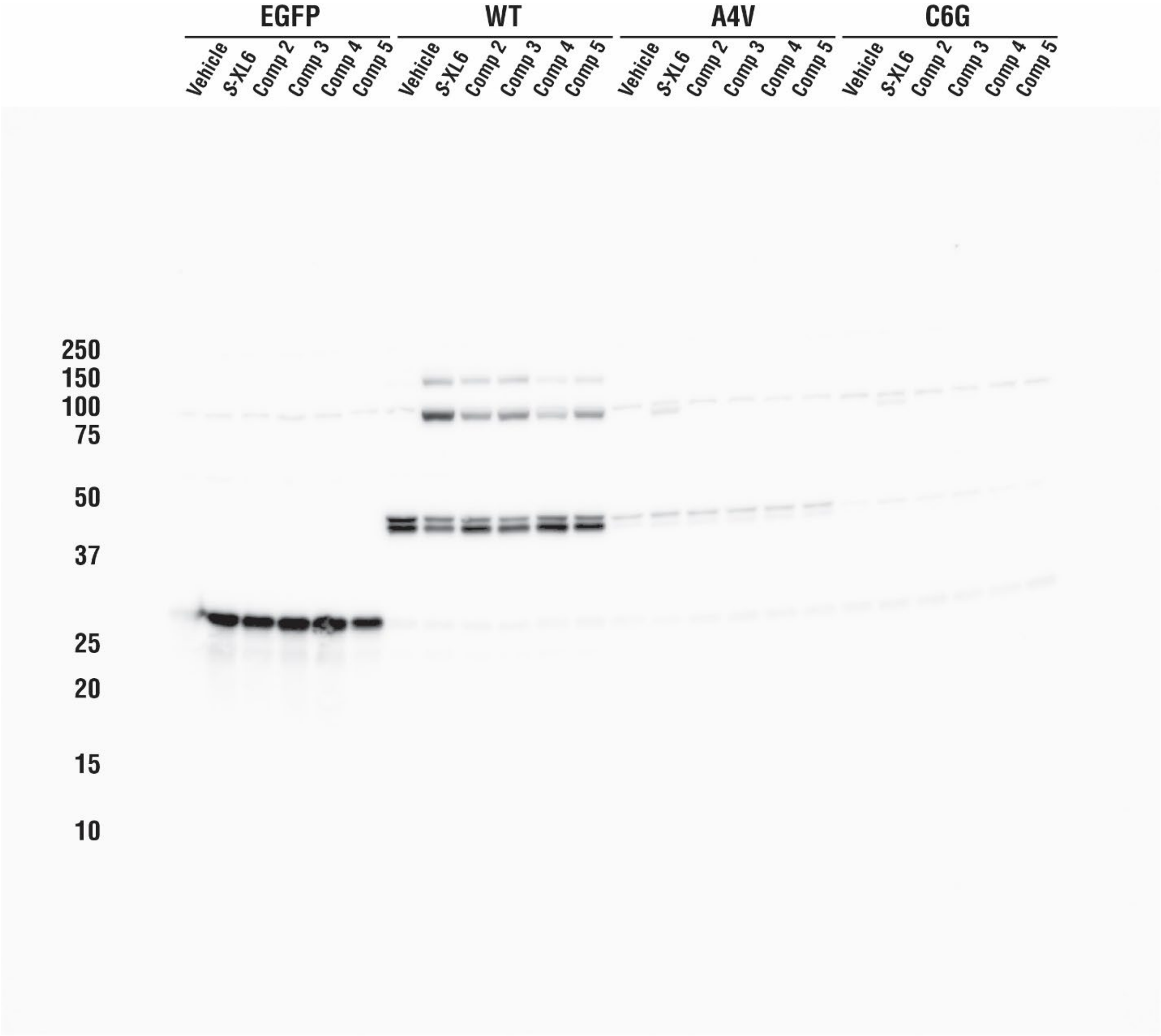
EGFP
WT
A4V
C6G
Vehicle
S-XL6
Comp 2
Comp 3
Comp 4
Comp 5
250
150
100
75
50
37
25
20
15
10

Fig. 7

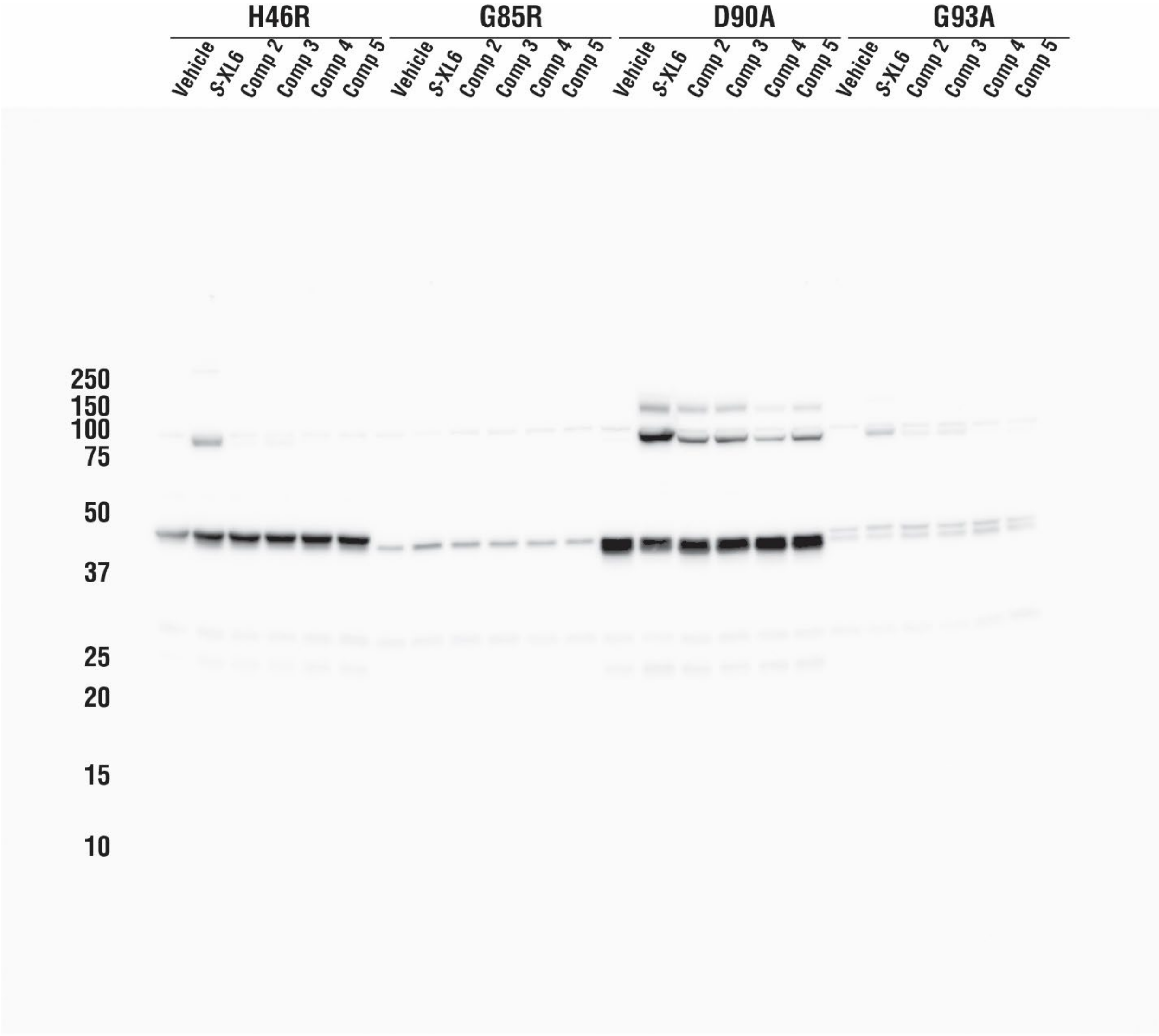
H46R
G85R
D90A
G93A
Vehicle
S-XL6
Comp 2
Comp 3
Comp 4
Comp 5
250
150
100
75
50
37
25
20
15
10

Fig. 7

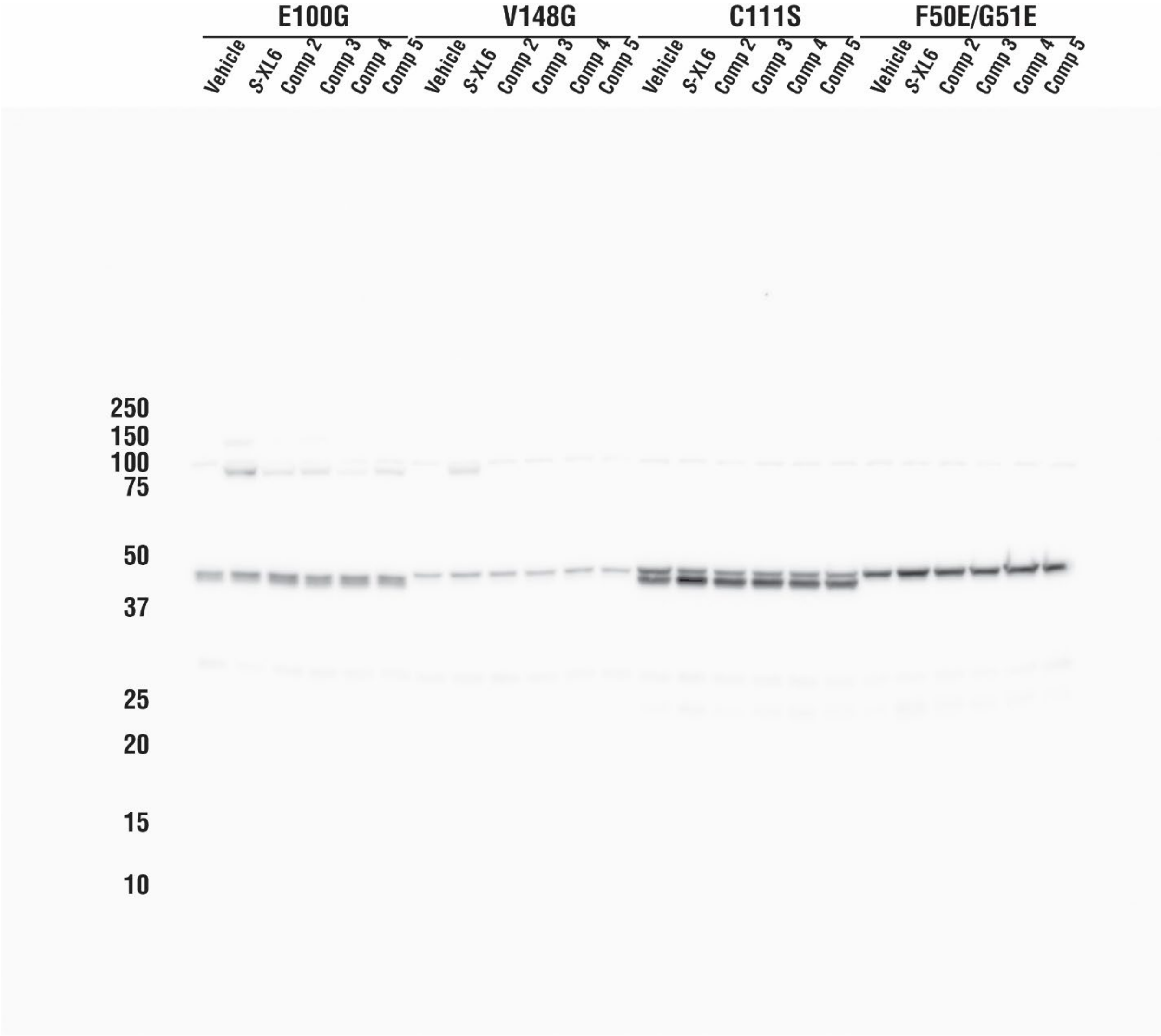
E100G
V148G
C111S
F50E/G51E
Vehicle
S-XL6
Comp 2
Comp 3
Comp 4
Comp 5
250
150
100
75
50
37
25
20
15
10

Fig. 7
